# Modeling host-microbiome interactions for the prediction of meat quality and carcass composition traits in swine

**DOI:** 10.1186/s12711-020-00561-7

**Published:** 2020-07-29

**Authors:** Piush Khanal, Christian Maltecca, Clint Schwab, Justin Fix, Matteo Bergamaschi, Francesco Tiezzi

**Affiliations:** 1 Department of Animal Science, North Carolina State University, Raleigh, NC 27695 USA; 2 The Maschhoffs LLC, Carlyle, IL 62231 USA

## Abstract

**Background:**

The objectives of this study were to evaluate genomic and microbial predictions of phenotypes for meat quality and carcass traits in swine, and to evaluate the contribution of host-microbiome interactions to the prediction. Data were collected from Duroc-sired three-way crossbred individuals (n = 1123) that were genotyped with a 60 k SNP chip. Phenotypic information and fecal 16S rRNA microbial sequences at three stages of growth (Wean, Mid-test, and Off-test) were available for all these individuals. We used fourfold cross-validation with animals grouped based on sire relatedness. Five models with three sets of predictors (full, informatively reduced, and randomly reduced) were evaluated. ‘Full’ included information from all genetic markers and all operational taxonomic units (OTU), while ‘informatively reduced’ and ‘randomly reduced’ represented a reduced number of markers and OTU based on significance preselection and random sampling, respectively. The baseline model included the fixed effects of dam line, sex and contemporary group and the random effect of pen. The other four models were constructed by including only genomic information, only microbiome information, both genomic and microbiome information, and microbiome and genomic information and their interaction.

**Results:**

Inclusion of microbiome information increased predictive ability of phenotype for most traits, in particular when microbiome information collected at a later growth stage was used. Inclusion of microbiome information resulted in higher accuracies and lower mean squared errors for fat-related traits (fat depth, belly weight, intramuscular fat and subjective marbling), objective color measures (Minolta a*, Minolta b* and Minolta L*) and carcass daily gain. Informative selection of markers increased predictive ability but decreasing the number of informatively reduced OTU did not improve model performance. The proportion of variation explained by the host-genome-by-microbiome interaction was highest for fat depth (~ 20% at Mid-test and Off-test) and shearing force (~ 20% consistently at Wean, Mid-test and Off-test), although the inclusion of the interaction term did not increase the accuracy of predictions significantly.

**Conclusions:**

This study provides novel insight on the use of microbiome information for the phenotypic prediction of meat quality and carcass traits in swine. Inclusion of microbiome information in the model improved predictive ability of phenotypes for fat deposition and color traits whereas including a genome-by-microbiome term did not improve prediction accuracy significantly.

## Background

Carcass composition and meat quality are economically important traits in the pig industry but, until recently, they have not been the major objectives in breeding programs [1, 2]. In addition to genetics, meat quality and carcass traits are affected by various other factors such as nutrition and environment [3–5]. Thus, to improve meat and carcass traits, it is necessary to have a good knowledge of the factors that influence a pig’s performances. Recently, most of the efforts have been devoted to exploiting the genomic variability of pigs for selection purposes [6–8]. However, the gut microbiome is a key component of all mammals and contributes significantly to the variation of many phenotypes [9]. Microbial communities are responsible for a large variability across a wide array of phenotypes, and the number of genes in the microbiome (often referred to as the second genome) is twice the number of genes in the host genome [10]. The effect of the variability of the host microbiome on carcass quality traits has been little explored in pork production.

The gastrointestinal tract of pigs contains a complex microbial ecosystem that interacts with the host and contributes to several of its biological functions, in particular functions related to health and well-being [11, 12]. Previous studies in humans [13, 14] have reported that variability of the microbiota should be accounted for to better understand health and disease. With the advent of efficient and cheap sequencing technologies, research on the role of the gut microbiome on animal health and performance has increased [15–17]. In swine, several studies [18, 19] have shown that the microbiome contributes significantly to the phenotypic variation in growth traits and suggested that microbiome composition could be a useful predictor of complex phenotypes.

In pigs, prediction of phenotypic performance of individuals is now routinely performed with the inclusion of genomic information but, to date, the advantage of including microbiome information has not been fully assessed. Recent studies [19–21] have reported estimates of the accuracy of microbial predictions for pig traits, but studies that include both genomic and microbiome information for the prediction of phenotypes are scarce. In particular, the effect of including microbial and host-microbiome interactions on the prediction of meat quality and carcass phenotypes has not been studied on a large scale and at multiple stages of the production life of the pig. Thus, the objectives of this study were: (i) to evaluate genomic and microbial predictions of phenotypes for meat quality and carcass traits, and (ii) to evaluate the effect of including a host-microbiome interaction for the prediction of these traits in swine. This study is a follow-up of the study by Khanal et al. [22] that used data from the same experiment. In this paper, we expanded the results on the estimates of microbiability and microbial correlations among carcass quality traits by focusing on prediction of carcass quality with the inclusion of genomic and microbial information as well as their interaction. In addition, we explored the effect of feature preselection, both genomic and microbial, on explained variance as well as prediction performance.

## Methods

Animal welfare approval was not needed for this study since all the data came from animals that were raised in a commercial setting by The Maschhoffs LLC (Carlyle, IL, USA) under routine conditions. All pigs were harvested in commercial facilities under the supervision of USDA Food Safety and Inspection Service.

### Animals and sample collection

The pigs included in this study consisted of 1123 three-way crossbred individuals obtained from 28 purebred Duroc sires and 747 commercial Yorkshire × Landrace or Landrace × Yorkshire F_1_ sows. The pigs were weaned at 18.6 ± 1.1 days of age and moved to a nursery-finishing facility. Weaned pigs were kept in single-sire single-sex pens with 20 pigs per pen. The test period began on the day the pigs were moved to the nursery-finishing facility. During the nursery, growth, and finishing periods, pigs were fed a standard pelleted feed depending on sex and age. The diet and their nutritional values are in Additional file 1: Table S1 (see Additional file 1: Table S1). Standard vaccination and medication protocols were followed (see Additional file 2: Tables S2, S3, and S4). The end of test was reached on a pen basis when the average weight of the pigs in the pen reached 138 kg. Rectal swabs were collected from all pigs at three stages of production (referred to “stage” hereafter): weaning (Wean), 15 weeks post-weaning (Mid-test; average 118.2 ± 1.18 days), and Off-test (Off-test, average 196.4 ± 7.80 days). Rectal swabs from four to five pigs per pen were used for subsequent microbial sequencing. These pigs were selected as described by Wilson et al. [23] to represent an average pig for body weight, along with pigs with a body weight that was approximately 1 and 2 SD above and below the pen average. This resulted in data on 1205, 1295 and 1273 samples for Wean, Mid-test, and Off-test, respectively. The distribution of these samples across families, growth stages and sex are in Additional file 3: Table S5. The following meat quality traits were measured on each pig, as described by Khanal et al. [6]: intramuscular fat content (IMF), Minolta a* (MINA), Minolta b* (MINB), Minolta L* (MINL), ultimate pH (PH), subjective color score (SCOL), subjective marbling score (SMARB), subjective firmness score (SFIRM), and shearing force (SSF)]. Minolta L*, a* and b* color scores measured the lightness (greater L* indicates a lighter as opposed to darker color), redness (greater a* indicates a redder color as opposed to green), and yellowness (greater b* indicates a yellower color as opposed to blue) and were recorded with a Minolta CR-400 Chroma meter (Minolta Camera Co. Ltd., Osaka, Japan). The following carcass composition traits were used for analysis: belly weight (BEL), ham weight (HAM), loin weight (LOIN), fat depth (FD), loin depth (LD), and carcass average daily gain (CADG). A summary of the traits included in the current research is in Table S6 [see Additional file 3: Table S6]. In total, data on 1123 individuals with complete genotypic, phenotypic and microbiome information at each stage were used for further analyses.

### Illumina amplicon sequencing

DNA extraction, purification, Illumina library preparation, and sequencing were done as described in [18]. Briefly, total DNA (gDNA) was extracted from each rectal swab by mechanical disruption in a phenol: chloroform: isoamyl alcohol (25:24:1, pH 8.0) solution. DNA was purified using the QIAquick 96 PCR purification kit (Qiagen, MD, USA) according to the manufacturer’s recommendations but with the following minor modifications: (i) sodium acetate (3 M, pH 5.5) was added to Buffer PM to a final concentration of 185 mM to ensure optimal binding of genomic DNA to the silica membrane; (ii) crude DNA was combined with 4 instead of 3 volumes of Buffer PM; and (iii) DNA was eluted in 100 instead of 80 µL Buffer EB. All sequencing was performed on an Illumina MiSeq instrument (Illumina, Inc. San Diego, USA), generating 250-bp paired-end reads, at the DNA Sequencing Innovation Laboratory at the Center of Genome Sciences and Systems Biology at Washington University in St. Louis. Phased, bi-directional amplification of the v4 region (515–806) of the 16S rRNA gene was carried out to generate indexed libraries for Illumina sequencing, as described in Faith et al. [24].

## 16S rRNA gene sequencing and data quality control

First, pairs of 16S rRNA gene sequences were merged into a single sequence using FLASH v1.2.11 [25] with a required overlap of at least 100 and less than 250 bp in order to provide confident overlaps. Then, sequences with a mean quality score lower than Q35 were filtered out using PRINSEQ v0.20.4 [26]. Sequences were oriented in the forward direction and reads that matched to any primer sequences (number of tolerated mismatches = 1 bp) were trimmed off. Sequences were subsequently demultiplexed using QIIME v1.9 [27]. Sequences with more than 97% nucleotide sequence similarity with the 16S rRNA sequence were clustered into operational taxonomic units (OTU) using QIIME with the following settings: max_accepts = 50, max_rejects = 8, percent_subsample = 0.1 and -suppress_step4. A modified version of Green Genes [28] was used as reference database. Ninety percent of the input sequences were matched to the reference database, while the remaining 10% were clustered de novo with UCLUST [29] to generate new reference OTU. Then, the 90% of reads that matched with the reference database were re-assigned to this new reference OTU. The most abundant sequence in each cluster was used as representative sequence for the OTU. Sparse OTU were then filtered out by requiring a minimum total observation count of 1200 for an OTU to be retained (column-wise sum or raw count table), and the resulting OTU table was rarefied to 10,000 counts per sample. After data processing and quality control, 1755 OTU remained for further analyses.

### Genotyping

All pigs were genotyped with the PorcineSNP60 v2 BeadChip (Illumina, Inc., San Diego, CA). Quality control procedures were applied by removing single nucleotide polymorphisms (SNPs) with a call rate lower than 0.90 and a minor allele frequency lower than 0.05. After quality control, 42,529 SNPs remained for further analyses.

### Estimation of variance components, heritability, and microbiability

**Fig. 1:**
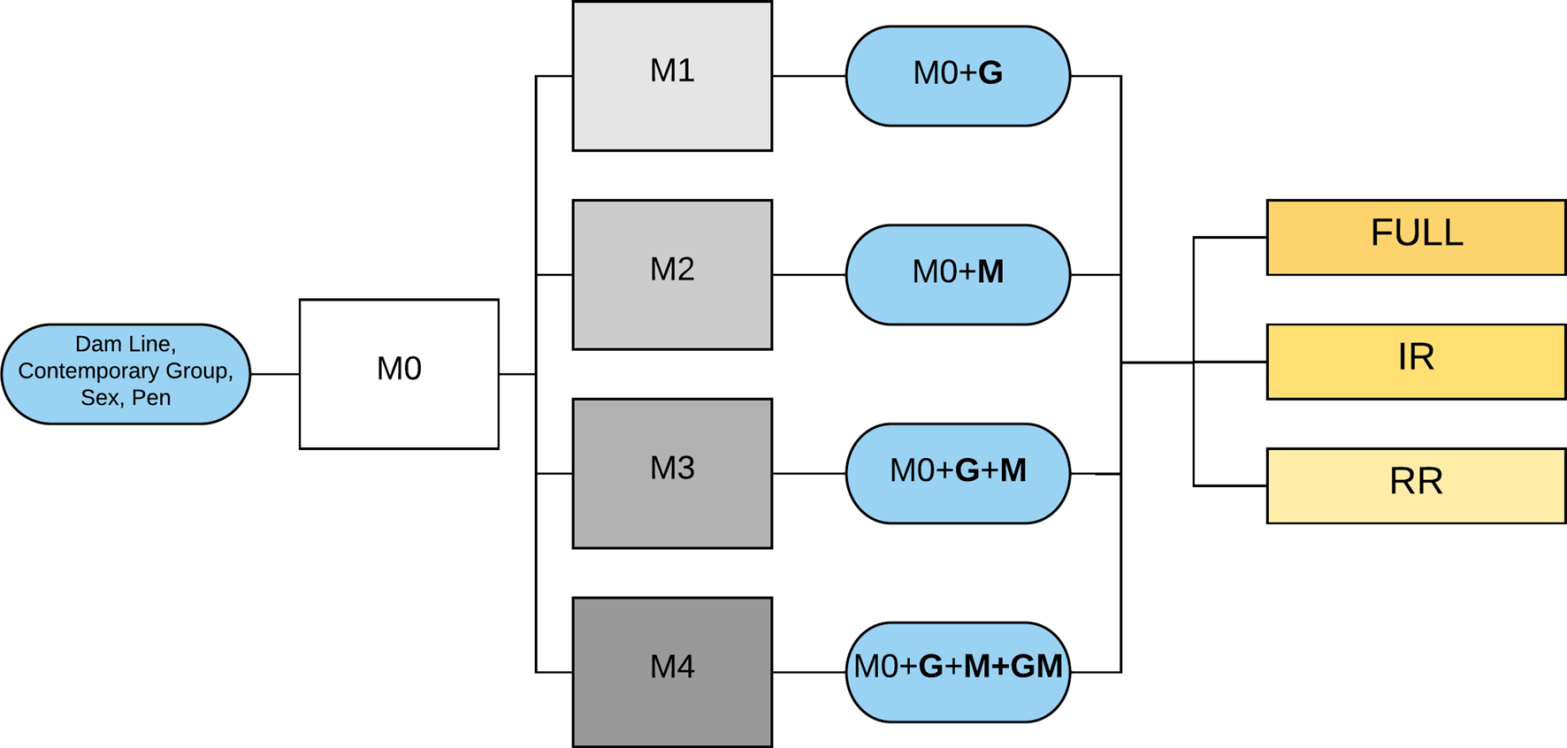
Overall design of the analyses. FULL contains all available markers and operational taxonomic units (OTU), IR contains informatively reduced markers and OTU, and RR contains randomly reduced markers and OTU. Compared to the base line model M0, M1 includes genomic information, M2 includes microbiome information, M3 includes microbiome and genomic information, and M4 includes genomic, microbiome and genome-by-microbiome interaction information

Several linear mixed models were used to analyze the trait phenotypes, as summarized in Fig. 1. The baseline model (M0) was defined as:

1 $$y_{ijklm} = \mu + dl_{i} + cg_{j} + sex_{k} + pen_{l\left( j \right)} + e_{ijklm} .$$

where $y_{ijklm}$ is the trait measured, $\mu$ the overall mean, $dl_{i}$ the fixed effect of the $i$th dam line (2 levels), $cg_{j}$ the fixed effect of the $j$th contemporary group (6 levels), $sex_{k}$ the fixed effect of sex $k$ (2 levels), $pen_{l\left( j \right)}$ the random effect of the $l$th pen nested within contemporary group, and $e_{ijklm}$ is the residual. Pen and residual effects were assumed normally distributed with mean zero and variances ${\mathbf{I}}\sigma_{pen}^{2}$ and ${\mathbf{I}}\sigma_{e}^{2} ,$ respectively, where ${\mathbf{I}}$ is an identity matrix, and $\sigma_{pen}^{2}$ and $\sigma_{e}^{2}$ are the pen and the residual variances.

### Model M1 was defined as

2 $$y_{ijklmn} = \mu + dl_{i} + cg_{j} + sex_{k} + pen_{l\left( j \right)} + a_{m} + e_{ijklmn}$$

where $a_{m}$ is the random additive genetic effect of animal $m$, which was assumed to be normally distributed with mean 0 and variance ${\mathbf{G}}\sigma_{a}^{2}$, where $\sigma_{a}^{2}$ is the total genomic variance and ${\mathbf{G}}$ is the genomic relationship matrix built on marker information following Method 1 of VanRaden [30].

In model M2, microbiome information was added to M0 to estimate the variance that is contributed by the microbiome at each growth stage. For this purpose, OTU information expressed as relative abundance was collected into a matrix ${\mathbf{S}}$, with dimensions $n \times q$, where $n$ is the number of animals and $q$ the number of OTU. Each element of ${\mathbf{S}}$, $S_{ij}$, is the relative abundance of OTU $j$ in animal $i$ at a given growth stage. A constant of 0.001 was added to all elements to avoid 0 representation and to facilitate subsequent operations. Matrix ${\mathbf{S}}$ was used to calculate the elements of matrix $X$ with the same dimensions as:

$$X_{ij} \, = \,\frac{{\log \left( {S_{ij} } \right) - \log \overline{{S_{.j} }} }}{{sd\left( {\log S_{.j} } \right)}}.$$

where ${\mathbf{S}}_{.j}$ is the vector of the $j$th column of ${\mathbf{S}}$. Thus, ${\mathbf{X}}$ contained log-transformed, centered, and scaled relative abundance of OTU. The microbial relationship matrix ${\mathbf{M}}$ was then created as a Gaussian kernel (equivalent to a genomic relationship matrix ${\mathbf{G}}$) as ${\mathbf{M}} = \frac{1}{q}{\mathbf{XX}}^{T}$, representing the covariance between individuals based on the resemblance of microbiome composition. Based on this, Model M2 was defined as:

3 $$y_{ijklmn} = \mu + dl_{i} + cg_{j} + sex_{k} + pen_{l\left( j \right)} + o_{m} + e_{ijklmn} .$$

and Model M3 as:

4 $$y_{ijklmn} = \mu + dl_{i} + cg_{j} + sex_{k} + pen_{l\left( j \right)} + a_{m} + o_{m} + e_{ijklmn} .$$

where $o_{m}$ is the random microbiome effect of animal $m$ at a given stage, as determined by its microbiome, with mean 0 and variance ${\mathbf{M}}\sigma_{o}^{2}$, with $\sigma_{o}^{2}$ the estimated microbiome variance.

Modeling all possible interactions between markers and OTU as independent covariates is computationally prohibitive. However, interactions between markers and OTU can be also modeled through covariance functions ${\mathbf{GM}}$ based on the Hadamard (cell-by-cell) product between ${\mathbf{G}}$ and ${\mathbf{M}}$; as shown by Jarquin et al. [31], the matrix resulting from the Hadamard product operation models the covariance between the two effects and thus can be used to fit the interaction effect in the model. The biological assumption behind this interaction is that allele substitution effects at each SNP depend on the gut microbial composition and, conversely, that OTU effects depend on the genotype of the host. Statistically, this interaction implies that resemblance between records is due to resemblance at both the host genomic and gut microbial levels. Note that these models closely resemble those commonly used to model genotype-by-environment interactions based on reaction norms [31], where in this case the gut microbiome composition is akin to the environmental component.

Based on this, Model M3 can be expanded to Model M4 as:

5 $$y_{ijklmn} = \mu + dl_{i} + cg_{j} + sex_{k} + pen_{l\left( j \right)} + a_{m} + o_{m} + ao_{m} + e_{ijklmn} .$$

where $ao_{m}$ is the random effect of animal $m$ as determined by the interaction between the additive genetic and microbiome effects $ao$, which was assumed to follow a normal distribution $ao\sim N(0,\left[ {{\mathbf{G}}^\circ {\mathbf{M}}} \right]\sigma_{ao}^{2}$, where $\sigma_{ao}^{2}$ is the estimated variance for this interaction and ${\mathbf{G}}^\circ {\mathbf{M}}$ indicates the Hadamard product of ${\mathbf{G}}$ and ${\mathbf{M}}$. In summary, the animal effect was accounted for by one deviation in Models M1 and M2, two deviations in M3, and three deviations in M4.

All analyses were performed using Bayesian Reproducing Kernel Hilbert Space (RKHS) regression models [32, 33], which offer a vast range of options, including higher non-linear interactions between terms by the use of different kernels. In this paper, for reasons of simplicity and to be able to compare our results directly with those of the literature, we used linear Gaussian kernels for ${\mathbf{G}}$, ${\mathbf{M}}$ and ${\mathbf{G}}^\circ {\mathbf{M}}$, which make the models directly comparable to best linear unbiased prediction (BLUP). Models were implemented in the BGLR package [34] in R, using a Markov chain Monte Carlo algorithm that was run for 120,000 iterations, with 20,000 iterations discarded as burn-in and a thinning interval of 5 iterations to estimate parameters as posterior means. Convergence of the models was checked by visual inspection of trace plots of variance components and by post-Gibbs analyses using the CODA package in R [35] (results not shown).

### Predictive ability of reduced models

To test the possibility of increasing the predictive ability of the models by using a reduced number of markers and OTU, all models presented in the previous section were run with three sets of predictors (referred to as “complexity” hereafter): full (FULL), informatively reduced (IR), and randomly reduced (RR). Full complexity corresponded to the use of all available markers and OTU to calculate ${\mathbf{G}}$ and ${\mathbf{M}}$, IR complexity to the use of only markers and OTU that were significantly associated with the trait, and RR complexity to the use of the same number (as for IR) of randomly sampled OTU and SNPs. The latter provided a better comparison with IR complexity.

Significance of the association of each marker/OTU with the trait phenotype was established using the following single marker linear regression model for trait phenotype with SNP allele count and OTU relative abundance as linear covariates:

6 $$y_{ijklmno} = \mu + dl_{i} + cg_{j} + sex_{k} + pen_{l\left( j \right)} + S_{m} + \beta X_{n} + e_{ijklmno} .$$

where $y,\mu, dl,cg,sex,pen$ are the same as above. *S*_*m*_ is the fixed effect $m$ of sires (28 levels), $X_{n}$ is the $n$th marker/OTU covariate, $\beta$ represents the marker/OTU effect, and all other terms were as described previously. The markers and OTU with a *P* value lower than 0.05 after Bonferroni correction, were considered significant (referred to “informatively reduced markers/OTU”, hereafter) and included in the calculation of ${\mathbf{G}}$ and ${\mathbf{M}}$ that were fitted in Models M1, M2, M3 and M4.

### Cross-validation

To evaluate predictive ability of the models, a stratified fourfold cross-validation scheme was used to split the data into training (~ 75% of observations) and testing (~ 25% of observations) sets, in which the individuals were grouped based on relatedness of the 28 sires in the trial. In each fold, progenies from 21 sires were allocated to the training set and progenies from the remaining seven sires were assigned to the testing set.

Different sets of markers and OTU were selected concomitantly with the four folds of the cross-validation scheme. For IR complexity, a unique set of significant markers was selected from each training set, and for growth a unique set of significant OTU was selected for each stage from each training set because the sets of OTU differed by growth stage. The numbers of significant markers and OTU and the numbers of common markers and OTU obtained from each training set are in Additional file 4: Tables S7 and S8 [see Additional file 4: Tables S7 and S8]. For RR complexity, a unique set of SNPs and OTU was randomly selected from each training set.

The predictive ability of each model was assessed by comparing the observed phenotype values ($y_{test}$) with the predicted phenotypes ($\hat{y}_{test}$) in the testing set using Pearson’s product-moment correlation coefficient and the mean squared error (MSE) of prediction, calculated as the average of squared differences between $y_{test}$ and $\hat{y}_{test}$. Raw phenotypes instead of the phenotypes adjusted for fixed effects were used to enable the use of M0 as a reference and to test the predictive ability of all effects independently.

### Post-analysis

In order to provide a comprehensive assessment of all factors in the experimental design, we conducted a post-analysis of the results by pooling predictive abilities for trait, growth stage, complexity, and model into a single dataset. The following linear model was then fitted:

7 $$\begin{aligned} y_{ijklmn} = T_{i} + S_{j} + M_{k} + C_{l} + F_{m} + SM_{jk} \hfill \\ \,\,\,\,\,\,\,\,\,\,\,\,\,\,\, + SC_{jl} + MC_{jk} + TS_{ij} + TM_{ik} + TC_{il} + e_{ijklmn} . \hfill \\ \end{aligned}$$

where $y_{ijklmn}$ is the prediction ability of each trait/stage/model/complexity/fold combination; $T_{i}$ the fixed effect of the traits measured (15 traits), $S_{j}$ the fixed effect of growth stage (3 levels: Wean, Mid-test and Off-test), $M_{k}$ the fixed effect of model (5 levels: M0, M1, M2, M3 and M4), $C_{l}$ the fixed effect of complexity of the model (3 levels: Full, IR and RR), $F_{m}$ the fixed effect of each fold of cross-validation (4 levels: Folds 1, 2, 3, and 4), while $SM_{jk}$, $SC_{jl}$, $MC_{jk}$, $TS_{ij}$, $TM_{ik}$ and $TC_{il}$ are pairwise interactions of the main effects. The ‘lm’ function of R was used to fit the model [36]. The type III ANOVA table was obtained from the ‘Anova’ function of the R package *car* [37] and lsmeans and pairwise contrasts were obtained from the *emmeans* [38] package in R.

## Results

### Data summary

Means and standard deviations for each of the nine meat quality and six carcass composition traits are in Additional file 3: Table S6. The distribution of taxonomic abundances at the three growth stages (Wean, Mid-test, Off-test) for these animals was previously reported by Lu et al. [18]. Briefly, across the three stages, there were 14, 21, 29, 54, 106, and 202 identified phyla, classes, orders, families, genera, and species, respectively, and 95.8 to 97.8% of the OTU were classified into six phyla: *Firmicutes*, *Bacteroidetes*, *Proteobacteria*, *Fusobacteria*, *Spirochaetes*, and *Actinobacteria*. *Firmicutes* represented the highest proportion of OTU, followed by *Bacteroides*. *Bacteroides* and *Firmicutes* together accounted for 73.6, 95.4, and 93.3% of all reads for the Wean, Mid-test, and Off-test stages, respectively.

### Variances explained by $G$, $M$ and their interaction

Estimates of variance components, heritabilities, and microbiabilities (proportion of variance explained by the microbiome) for the meat quality and carcass traits are in Additional file 5: Tables S9 and S10 [see Additional file 5: Tables S9 and S10]. Estimates of heritabilities and microbiabilities for these data were previously reported by Khanal et al. [22]. Briefly, the estimate of the proportion of variance explained by the microbiome was higher for carcass composition traits than for meat quality traits. Estimates of the microbiability were negligible at the Wean stage for both meat quality and carcass composition traits. Three meat quality traits had a significant (P < 0.05) estimate of microbiability at Mid-test, with estimates of 0.07 ± 0.03 for SMARB, 0.08 ± 0.03 for SFIRM and 0.10 ± 0.04 for MINB. At Off-test, four meat quality traits had a significant (P < 0.05) estimate of microbiability, with estimates of 0.06 ± 0.02 for IMF, 0.09 ± 0.04 for MINA, 0.11 ± 0.04 for MINB and 0.13 ± 0.04 for SFIRM. Most carcass traits were significantly (P < 0.05) affected by the microbiome at Mid-test and Off-test. The estimate of the microbiability of carcass traits at Mid-test ranged from 0.12 ± 0.04 for LOIN and FD to 0.20 ± 0.04 for BEL. The microbiability of carcass composition traits at Off-test ranged from 0.13 ± 0.05 for LOIN to 0.29 ± 0.05 for BEL. For most of the carcass traits, the additive genomic variance was eroded when microbiome information was included in the model, particularly at Mid-test and Off-test, which suggests a possible microbiome-host interaction. In this study, this component was included in the models explicitly.

**Fig. 2:**
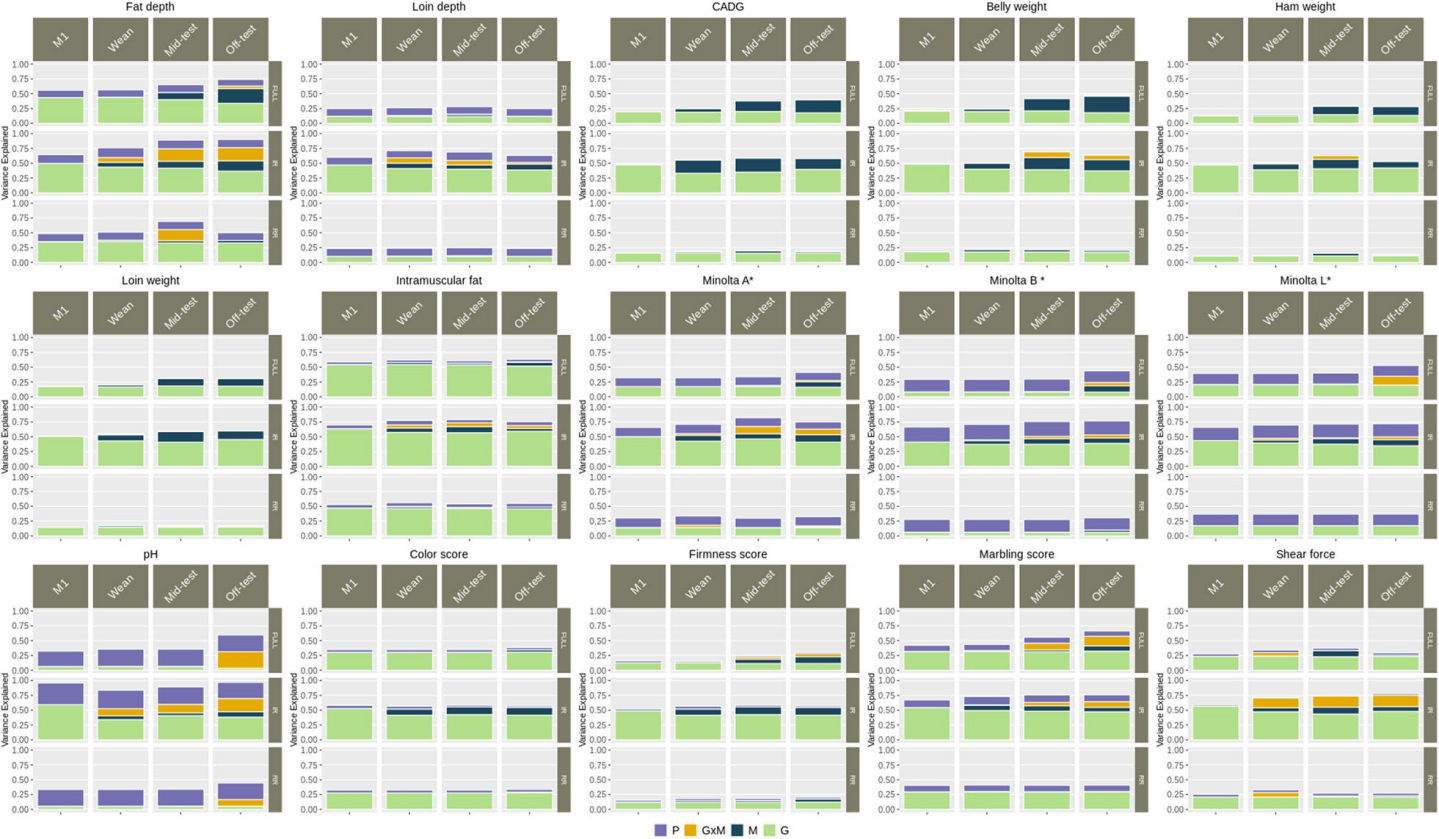
Variance component estimates for carcass and meat quality traits obtained from each model. Models M1, M2, M3 and M4 include only host genotype information, only gut microbiome information, host genotype and gut microbiome information, and host genotype, gut microbiome and genotype-by-microbiome interaction, respectively, at three stages of production (Wean, Mid-test and Off-test) with different sets of markers and operational taxonomic units (OTU relative abundance) (FULL: contains all available markers and OTU, IR: contains informatively reduced markers and OTU, and RR: contains randomly reduced markers and OTU). Each individual bar in the plot depicts the total variance of each model, partitioned by the proportion of variance explained by host genomic effect (*G*), gut microbiome effect ($M$), host genomic by gut microbiome ($G \times M$) and pen ($P$) effects

Estimate of variance components with the inclusion of interaction in the model at different stages are reported in Fig 2. With the inclusion of $ao$ in the model, there was no decrease in the genomic heritability compared to the model that contained genomic and microbiome information separately, except for PH and MINL for which the genomic heritability decreased by ~ 2% (Fig. 2).

For most traits, the proportion of variance explained by $ao$ was higher with IR than with FULL complexity. With IR complexity, the magnitude of the interaction was larger at Off-test, followed by Mid-test and Wean, except for LD, for which the trend was reversed, and the proportion of variance explained by $ao$ was highest at Wean and lowest at Off-test. Among the carcass composition traits, the proportion of variance explained by $ao$ was highest for fat depth (~ 20% at Mid-test and Off-test) and virtually null for CADG, HAM and LOIN. Among the meat quality traits, the proportion of variance explained by $ao$ was highest for SSF (~ 20% consistently across growth stages) and almost null for SCOL. With IR complexity, FD, PH, and SSF had a higher proportion of variance explained by $ao$ than the proportion of variance explained by the microbiome itself. About 10, 8 and 11% more variance was explained by $ao$ than by $o$ for SSF at Wean, Mid-test, and Off-test, respectively. Similarly, about 6, 10 and 13% more variance was explained by $ao$ than by $o$ for PH at Wean, Mid-test, and Off-test, respectively. About 10 and 5% more variance was explained by $ao$ than by $o$ for FD at Mid-test and Off-test, respectively. With RR complexity, the proportion of variance explained by $ao$ was almost null for most traits, as expected under the hypothesis that randomly selecting features will miss the signal of significant markers and OTU (Fig. 2).

### Predictive ability

**Fig. 3:**
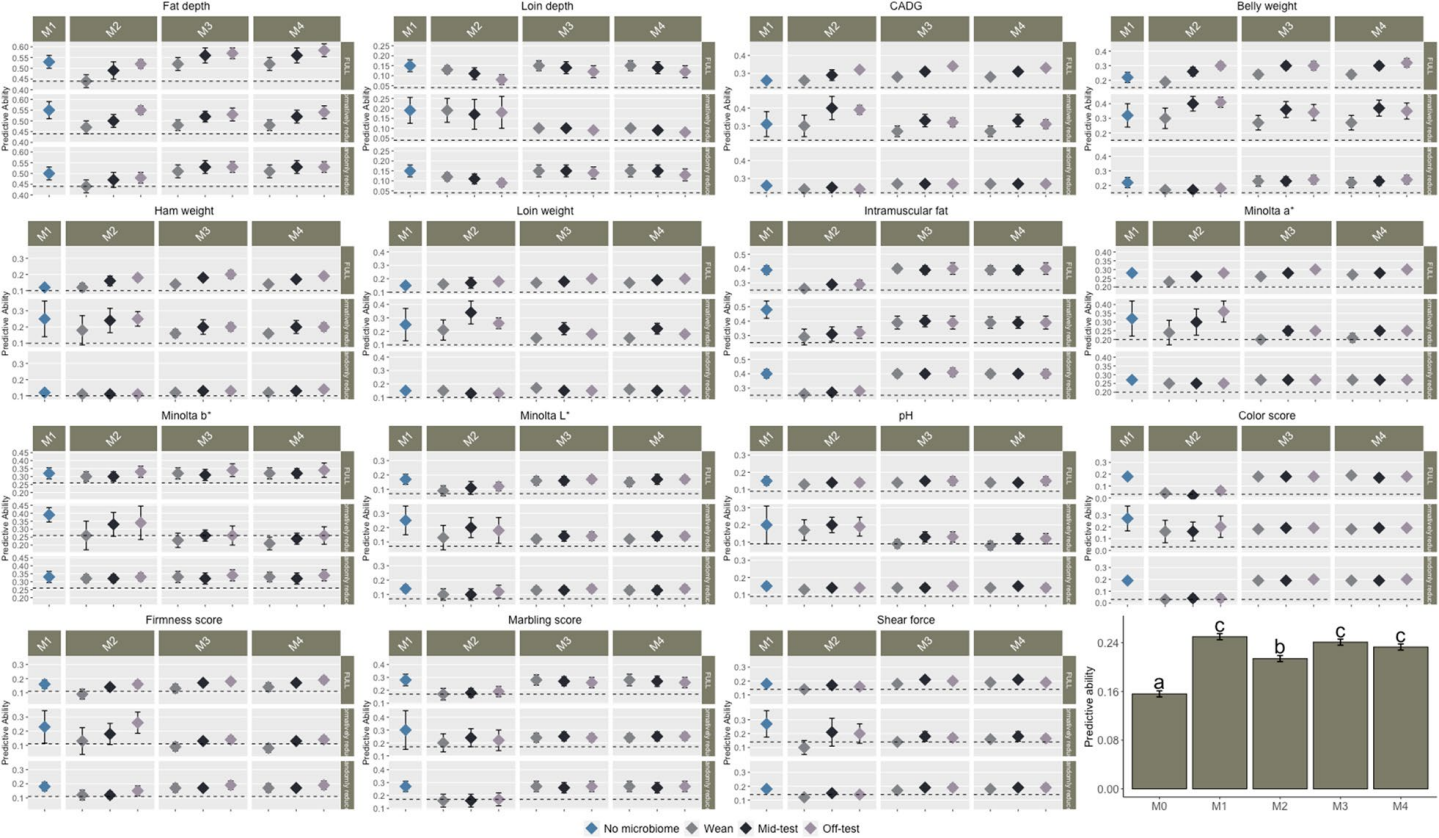
Predictive ability for carcass and meat quality traits. Each point corresponds to the mean predictive ability of a model with standard deviation, with different complexities (Full with all markers and operational taxonomic units (OTU), Informatively reduced with significant markers and OTU, and Randomly reduced with randomly selected markers and OTU). The last panel contains a summary of the prediction accuracies for all traits. Model M0 contains all fixed effects and only pen as random effect; M1 contains the animal genetic effect in addition to M0; M2 contains the microbiome effect at Wean, Mid-test and Off-test, in addition to M0. M3 contains the individual genomic as well as microbiome effects at Wean, Mid-test and Off-test, in addition to M0. M4 contains both the main effect of host genomic and microbiome effect as well as the interaction between them, in addition to M0

**Fig. 4:**
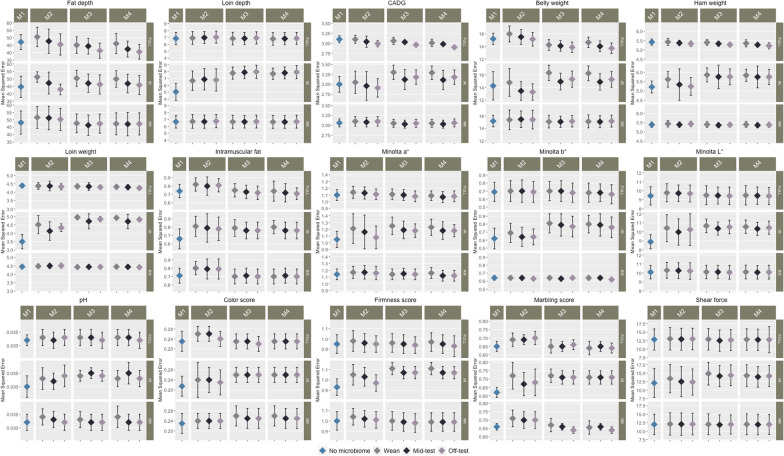
Mean squared error of prediction for carcass and meat quality traits. Each point shows the average mean squared error (MSE) for each model with standard deviation at different complexities (Full with all markers and operational taxonomic units (OTU), Informatively reduced with significant markers and OTU, and Randomly reduced with randomly selected markers and OTU). M1 contains the genomic relationship matrix. M2 contains the microbiome relationship matrix at Wean, Mid-test and Off-test. M3 contains the genomic relationship matrix and microbiome relationship matrix at Wean, Mid-test and Off-test. M4 contain both the main effect of the microbiome relationship matrix and genomic relationship matrix and the interaction between them

We investigated the effectiveness of various prediction models under different complexity levels (FULL, IR and RR). Results of this analysis and a summary of the predictive abilities for all traits are reported in Fig. 3. The predictive abilities for individual folds are in Additional file 6: Tables S11 to S25 [see Additional file 6 Tables S11 to S25]. The histograms in Fig. 3 represent the average predictive abilities and their respective standard deviation. Mean squared errors (MSE) of the models for FULL, IR and RR complexities are reported in Fig. 4.

With FULL complexity, inclusion of genomic and microbiome information resulted in greater predictive ability for most traits compared to the baseline model M0. The models that included only microbiome information (M2), both microbiome and genomic information (M3), and microbiome, genomic information and their interaction (M4) had greater predictive ability compared to the model that included only genomic information (M1) for most of the carcass traits, especially at Mid-test and Off-test. Model M2 outperformed M1 for BEL, CADG, HAM and LOIN, especially at Mid-test and Off-test with an average gain of approximately 4.5%. The predictive ability of Models M3 and M4 was greater than that of M1 for FD, BEL, CADG, HAM, LOIN, IMF, SSF, MINA and MINB. Among all the traits that had a greater predictive ability with Model M3 compared to M1, BEL showed the largest increase in prediction ability (~ 7% averaged over all production stages). Similarly, BEL showed the largest increase in predictive ability with Model M4 compared to M1 (~ 7.5% averaged over all production stages). The predictive ability of Models M3 and M4 was greater than that of M2 for most traits. The largest increase in predictive ability for M3 and M4 compared to M2 was ~ 14% (averaged over each stage) for SCOL. The predictive power of the models that included microbiome information (M2, M3 and M4) was substantially larger for most of the traits at Mid-test and Off-test than at Wean. The predictive ability of M4 did not improve significantly compared to M3. In terms of MSE, for FD, CADG, HAM, IMF and MINB, models that included the interaction between genomic and microbiome information (M4) at Off-test had lower MSE compared to the other models.

As with FULL complexity, with IR complexity, inclusion of genomic and microbiome information resulted in higher predictive power compared to the null model M0. Model M2 outperformed M1 for BEL, CADG and LOIN at Mid-test and Off-test by approximately 8% across trait/time combination. The predictive ability was comparable for models that contained microbiome or genomic information (M1 and M2). However, including genomic and microbiome information together (M3 and M4), decreased the predictive ability for all traits, except for BEL for which the predictive ability of M3 and M4 was greater than that of M1 at Off-test and at Mid-test by ~ 3 and ~ 5%, respectively. M3 and M4 performed similarly for all meat quality and carcass traits.

Preselection of markers and OTU increased the predictive ability of M1 and M2 for all traits compared to FULL complexity. The MSE of M1 with IR complexity was smaller compared to M1 with FULL complexity. However, the MSE of M2 with IR complexity was greater compared to that of M2 with FULL complexity for most traits (Fig. 4). For most traits, models that included both genomic and microbiome information and the genome-by-microbiome interaction did not perform better in terms of predictive ability. Based on MSE, models with IR complexity had greater MSE compared to those with FULL complexity. Randomly selecting markers and OTU resulted in a lower predictive ability and in higher MSE, as expected.

Based on the summary of the results, as expected, all models performed better than the baseline model M0. The predictive ability of models that included genomic information only was better than models that included microbiome information only (averaged over all other factors) but similar to models that included both microbiome and genomic information. However, predictive ability of the model that included only microbiome information was better for some traits (FD, BEL, CADG and LOIN). Averaging over all factors, Models M1, M3, and M4 performed similarly.

### Post-analysis

To evaluate the overall influence of all the factors in the experimental design on the predictive ability of the model, we conducted a post-analysis of the cross-validation study. Results of this analysis are in Table 1. All factors were highly significant (*P *< 0.001), except the interaction between Stage and Trait. The least square means of the significant main effects and their interactions are in Additional file 7: Tables S26−S30 [see Additional file 7: Tables S26–S30]. In brief, averaging over all factors, Models M1, M3, and M4 performed similarly. The predictive ability of models that included information recorded at Mid-Test and Off-test stages was higher than the predictive ability of models that included information recorded at Weaning. Feature selection (averaged across all the other terms) resulted in better predictive abilities than FULL and RR complexities. The best-predicted traits were the fat-related traits (FD, IMF, and BEL) and CADG, regardless of the model used, whereas the least accurately predicted traits were LD, MINL, SCOL and PH. The results for the interaction between Stage and Model showed that at Mid-test and Off-test, Models M1, M3 and M4 performed similarly, but at weaning, M1 performed better than M3 and M4. The results for the interaction between Complexity and Model showed that Model M1 with IR complexity had a high predictive performance, while the baseline model (M0) had the worst predictive performance at all production stages.

**Table 1:** Type III ANOVA of post-analysis of the experimental design

	Sum square	Mean square	F value	P- value
Complexity	0.49	0.25	57.94	< 0.001
Complexity:Trait	0.40	0.01	3.35	< 0.001
Fold	1.43	0.48	112.14	< 0.001
Model	3.08	0.77	181.44	< 0.001
Model:Complexity	1.03	0.13	30.50	< 0.001
Model:Stage	0.12	0.02	3.57	< 0.001
Model:Trait	1.45	0.03	6.09	< 0.001
Stage	0.12	0.06	13.88	< 0.001
Stage:Complexity	0.05	0.01	2.83	0.023
Stage:Trait	0.09	0.00	0.80	0.77
Trait	26.66	1.90	449.20	< 0.001

Model (5 levels: M0, M1, M2, M3 and M4), Stage (3 levels: Wean, On test and Off test), Complexity (3 levels: Full, IR (Informatively reduced), RR (Randomly reduced), Trait (15 levels: FD, CADG, LD, HAM, LOIN, BEL, IMF, SMARB, SCOL, MINA, MINB, MINL, pH, SSF, SFIRM). All elements with (:) represent pairwise interactions

## Discussion

Previous studies have explored the host-microbiome interplay in swine [39, 40]. However, these studies neither included genomic information of the host, nor fitted an explicit interaction between host and microbiome components. In the current study, we evaluated the effect of the host genome, gut microbiome, and their interaction on the phenotypic prediction of meat quality and carcass traits in swine. For this purpose, we used various models with different sets of SNPs and OTU to provide a better understanding of the contribution of each component to the predictive ability.

### Genomic-by-microbiome interaction

For most of the meat quality measures, the microbial variance contributed to the overall variability, but to a much lesser extent than for growth traits. Fang et al. [41] reported that gut bacteria were involved in energy metabolism and subsequently in the intramuscular fat content of muscle in pig, but our study showed a moderate impact of the microbiome on IMF. The host-genome-by-microbiome interaction ($ao$) showed a substantial contribution to the variance of several traits. The magnitude of the variance of the genome-by-microbiome interaction was sizable for fat-related traits (FD, BEL, SMARB, and IMF) but small for objective color traits (MINA, MINB, and MINL), PH and SSF. In humans, a significant contribution of the interaction between the host genetics and the gut microbiome to obesity has been demonstrated [42–45]. Gut microbes produce short-chain fatty acids that regulate the host body energy homeostasis [46, 47] and deposition of fat in body and muscle. The existence of a sizable portion of the phenotypic variance in swine growth that can be explained by the microbiome alone, as well as by a genome-by-microbiome interaction, represents both a challenge and an opportunity for selection programs. From a breeding perspective, we can expect that if the interactions between host and microbiome are heritable, individuals will re-rank based on their genomic value across different microbial compositions, and thus that some of the growth and carcass traits could be regulated by a slightly different gene set under different microbial conditions. This means that without the explicit inclusion of microbial information in the model used under these conditions, the selection response might be slightly hampered. Further research would be needed to elucidate this aspect, but in this case, the results from our study could be used as a proof of concept that germplasms that are adapted to the particular gut flora resulting from particular environmental or dietary conditions could be used in selection programs. In addition to interaction effects that can exist between the host genome and the gut microbiome, one should also consider that the breeding value could include a microbiome-determined component, as reported by Weishaar et al. [48]. It is also important to note that in our experimental design, diet was kept constant and a uniform influence of the environmental conditions on the microbial composition of the individuals was assumed. Further studies should relax these assumptions and identify key microbiome shifts due to or associated to diet, environment, and management conditions that could be used to better tailor genomic resources to specific production systems.

### Predictive ability

In this study, predictive ability increased and MSE decreased for most of the models that included microbiome information. These results agree with those of Maltecca et al. [18] and Lu et al. [20], which suggest that the microbiome can be used as a biomarker to predict phenotype for growth and carcass traits in swine. Our results also show that the predictive ability is generally greater when microbiome data collected at Off-test is included compared to earlier growth stages, although information collected at earlier stages would prove more useful in selection and management. The higher predictive ability obtained when including later-stage microbiome information is again in agreement with Maltecca et al. [18] and Lu et al. [20]. The explicit inclusion of a genome-by-microbiome interaction resulted only in marginal gains in accuracy for most traits, and in some cases, it resulted in a decrease in predictive ability. However, it should be noted that, in several cases, the MSE was lower for the model that included all the effects plus their interaction. Gonzalez-Recio et al. [49] reported that MSE is a preferred measure to select models because it considers both prediction bias and variability, whereas the predictive ability provides only a measure of association. In our study, the MSE results suggest that models that include a genome-by-microbiome interaction would be suitable for several growth and carcass phenotypes. In addition, it should be noted that including such an interaction would increase its predictive advantage as the cohorts used become larger [50].

### Feature selection

Selecting markers and OTU based on the association with the phenotypes resulted in a general increase of the contribution of genomic, microbial, and interaction terms to the total variance. This increase in the explained variance did not translate, for the most part, in a significant increase in predictive ability. More specifically, informatively reduced markers showed higher prediction accuracy compared to a whole or random set of SNPs. Previous studies have reported discordant results concerning this issue. While our results are in agreement with studies that achieved higher predictive performance by using an informatively-reduced set of markers [51, 52], other studies have found that a subset of markers does not improve or match the predictive performance achieved by the whole marker panel [53]. In contrast, selecting OTU often resulted in worse performances compared to the use of the whole set of predictors except for BEL, which could be due to the contribution of fewer OTU to belly weight. Several reasons may explain the worst performance of the model with IR complexity: (1) it is likely that the sample size of the current study was not sufficient to allow us to identify effectively significant taxa; (2) the analysis used (a simple linear regression) possibly did not effectively account for the compositional nature of microbiome data; and (3) each individual taxon may have a small contribution to the overall microbial effect, while some OTU may still show moderately stronger effects. Camarinha-Silva et al. [19] reported similar findings for daily gain, feed intake and feed conversion ratio in pigs, using a limited set of individuals, and suggested the term ‘polymicrobial’ for the overall contribution of the microbiome to phenotypic variation. Vollmar et al. [54], in a study conducted on Japanese Quail (*Coturnix japonica*), also concluded about the polymicrobial nature of some traits but showed that some OTU contributed more than others to the overall phenotypic variance. Our results seem to be in agreement with the mentioned studies and support the hypothesis of a polymicrobial effect with a larger contribution from some taxa, since feature selection of OTU seemed to perform better than random selection. Further studies that aim specifically at understanding the architecture of microbial variation are necessary to provide additional information on this issue.

## Conclusions

We conducted this study to investigate the effect of host-microbiome interactions on meat quality and carcass composition traits in swine at different growth stages (Wean, Mid-test and Off-test). To the best of our knowledge, this is the first attempt to investigate the effect of host-microbiome interaction on meat quality and carcass traits. Inclusion of microbiome information in the model increased the prediction ability for most traits, although this differed between growth stages. Microbiome information collected at a later stage of life led to better predictive ability. With the models that included microbiome information, predictive ability was higher for fat-related traits (fat depth, belly weight, intramuscular fat and subjective marbling), objective color measures (Minolta a*, Minolta b* and Minolta L*) and carcass daily gain. Informatively selected SNPs resulted in better predictive ability but reducing the number of OTU did not improve prediction ability.

## Supplementary information

**Additional file 1: Table S1.** Diet formulae and their nutritional values.

**Additional file 2: Table S2.** Vaccinations**. Table S3.** Injectable medications**. Table S4.** Water medications.

**Additional file 3: Table S5.** Distribution of samples across families, sex, and time points**. Table S6.** Descriptive statistics of carcass composition and meat quality traits.

**Additional file 4: Table S7.** Number of significant OTU of each fold of cross-validation and the number of common OTU for each trait at each stage within each fold. **Table S8.** Number of significant markers of each fold of cross-validation and the number of common markers for each trait at each stage within each fold.

**Additional file 5: Table S9.** Variance components explained by microbiome relationship matrix ($\bf O$), genomic relationship matrix ($\bf G$), pen ($\bf P$), residual ($\bf R$), microbiability (m^2^) and heritability (h^2^) in different models**. Table S10.** Variance components explained by microbiome relationship matrix ($\bf O$), pen ($\bf P$), residual ($\bf R$) and microbiability (m^2^) at different stages of production when only microbiome information was included in the model.

**Additional file 6: Table S11.** Predictive ability of Loin depth for each fold at different complexities and stages and with different models. **Table S12.** Predictive ability of fat depth for each fold at different complexities and stages and with different models. **Table S13.** Predictive ability of carcass average daily gain for each fold at different complexities and stages and with different models. **Table S14.** Predictive ability of ham weight for each fold at different complexities and stages and with different models. **Table S15.** Predictive ability of loin weight for each fold at different complexities and stages and with different models. **Table S16.** Predictive ability of belly weight for each fold at different complexities and stages and with different models. **Table S17.** Predictive ability of intramuscular fat (%) for each fold at different complexities and stages and with different models. **Table S18.** Predictive ability of Minolta a* for each fold at different complexities and stages and with different models. **Table S19.** Predictive ability of Minolta b* for each fold at different complexities and stages and with different models. **Table S20.** Predictive ability of Minolta L* for each fold at different complexities and stages and with different models. **Table S21.** Predictive ability of pH for each fold at different complexities and stages and with different models. **Table S22.** Predictive ability of subjective color score for each fold at different complexities and stages and with different models. **Table S23.** Predictive ability of subjective marbling score for each fold at different complexities and stages and with different models. **Table S24.** Predictive ability of Subjective firmness score for each fold at different complexities and stages and with different models. **Table S25.** Predictive ability of slice shear force for each fold at different complexities and stages and with different models.

**Additional file 7: Table S26.** Least square means of prediction accuracy of all models averaging over all the traits**. Table S27.** Least square means of prediction accuracy of three stages of production averaging over all the traits**. Table S28.** Least square means of prediction accuracy of three complexities of model averaging over all the traits**. Table S29.** Least square means of prediction accuracy of interaction of stage and model averaging over all the traits**. Table S30.** Least square means of prediction accuracy of interaction of complexity and model averaging over all the traits.

## Data Availability

The data that support the findings of this study are available from MATATU, but restrictions apply to the availability of these data, which were used under license for the current study, and thus are not publicly available. However, the data are available from the authors upon reasonable request and with permission of MATATU.
