# Supplementary material for: Modeling host-microbiome interactions for the prediction of meat quality and carcass composition traits in swine
Source: Genet Sel Evol. 2020 Jul 29;52:41. doi: 10.1186/s12711-020-00561-7 (PMC7388461; doi:10.1186/s12711-020-00561-7)
Supplement: Supplementary file 2 — Additional file 2: Table S2. Vaccinations. Table S3. Injectable medications. Table S4. Water medications. [file 12711_2020_561_MOESM2_ESM.pdf]

Table S2. Vaccinations

Condition	Timing
Mycoplasma Hyopneumoniae	Processing (~4 days old)
Porcine Circovirus Type 2 (PCV2)	Weaning
Porcine Respiratory Syndrome (PRRS)	10-14 days post-weaning
Porcine Circovirus<br>Mycoplasma Hyopneumoniae	10-17 days post PRRS vaccination
Ileitis<br>Erysipelas	~6 weeks post-weaning

Table S3. Injectable medications

Condition	Product	Timing
Respiratory, Diarrhea, Lameness	Excede	Weaning to 8 weeks post-weaning
Respiratory	Biomycin 200	8-14 weeks post-weaning
Respiratory	Lincocin 300	14 weeks post-weaning to end of study
Diarrhea, Lameness	Lincocin 300	8 weeks post weaning to end of study
Lameness	Dexamethasone	Weaning to 14 weeks post-weaning

Table S4. Water medications

Diarrhea	Product	Timing
Respiratory, Diarrhea	Neomycin	Weaning
Respiratory, Diarrhea, Lameness	Oxytetracycline (OTC)<br>Denagard	As needed
	Linco Soluble	As needed
