# Supplementary material for: Modeling host-microbiome interactions for the prediction of meat quality and carcass composition traits in swine
Source: Genet Sel Evol. 2020 Jul 29;52:41. doi: 10.1186/s12711-020-00561-7 (PMC7388461; doi:10.1186/s12711-020-00561-7)
Supplement: Supplementary file 3 — Additional file 3: Table S5. Distribution of samples across families, sex, and time points. Table S6. Descriptive statistics of carcass composition and meat quality traits. [file 12711_2020_561_MOESM3_ESM.pdf]

Table S5. Distribution of samples across families, sex, and time points

Family	Female			Male			Total
	Weaning	Mid-test	Off-test	Weaning	Mid-test	Off-test	
1	22	23	22	20	20	20	127
2	19	25	24	23	23	21	135
3	20	23	22	20	23	23	131
4	23	24	23	21	23	23	137
5	21	18	21	15	15	15	105
6	21	24	24	23	25	25	142
7	18	22	21	19	20	19	119
8	20	25	25	23	24	23	140
9	21	24	25	25	25	26	146
10	22	25	25	23	24	22	141
11	21	22	23	24	24	23	137
12	20	21	20	20	22	22	125
13	23	25	24	21	23	21	137
14	24	26	25	22	21	21	139
15	23	23	23	25	25	24	143
16	19	24	23	24	25	25	140
17	19	20	21	22	23	23	128
18	23	23	22	23	23	23	137
19	22	26	26	20	19	19	132
20	22	25	22	24	26	20	139
21	18	21	21	18	19	19	116
22	21	25	23	23	22	24	138
23	19	23	21	19	22	20	124
24	22	25	25	23	24	23	142
25	24	27	27	20	23	23	144
26	22	24	25	23	23	24	141
27	23	26	27	21	24	24	145
28	24	26	25	25	20	23	143

Table S6. Descriptive statistics of carcass composition and meat quality traits

Trait	Acronym	Mean	SD
**Carcass traits**			
Loin depth, mm	LD	67.99	7.21
Back fat depth, mm	FD	22.07	5.24
Carcass average daily gain, g/day	CADG	552.9	73.9
Ham weight, kg	HAM	25.19	2.34
Loin Weight, kg	LOIN	20.01	1.88
Belly weight, kg	BEL	15.88	2.55
**Meat quality**			
Intramuscular fat, %	IMF	2.71	1.01
Minolta a*	MINA	3.77	1.16
Minolta b*	MINB	-0.16	0.87
Minolta L*	MINL	45.37	5.76
Ultimate pH	PH	5.64	0.22
Subjective color	SCOL	2.72	0.57
Subjective marbling	SMARB	3.10	0.91
Subjective firmness	SFIRM	3.05	1.04
Slice shear force, Kg	SSF	16.01	4.28
