# Supplementary material for: Modeling host-microbiome interactions for the prediction of meat quality and carcass composition traits in swine
Source: Genet Sel Evol. 2020 Jul 29;52:41. doi: 10.1186/s12711-020-00561-7 (PMC7388461; doi:10.1186/s12711-020-00561-7)
Supplement: Supplementary file 4 — Additional file 4: Table S7. Number of significant OTU of each fold of cross-validation and the number of common OTU for each trait at each stage within each fold. Table S8. Number of significant markers of each fold of cross-validation and the number of common markers for each trait at each stage within each fold. [file 12711_2020_561_MOESM4_ESM.pdf]

Table S7. Number of significant OTU of each fold of cross-validation and the number of common OTU for each trait at each stage within each fold

Traits[1]	Stage	Fold 1	Fold 2	Fold 3	Fold 4	Common
LD	Wean	108	80	245	229	25
	Mid-test	79	83	84	80	30
	Off-test	89	71	97	79	19
FD	Wean	179	82	80	93	25
	Mid-test	340	381	393	375	207
	Off-test	273	308	359	379	156
CADG	Wean	276	110	144	108	36
	Mid-test	186	221	240	199	117
	Off-test	204	223	222	272	88
BEL	Wean	290	122	120	118	39
	Mid-test	118	297	264	129	46
	Off-test	182	266	267	279	99
HAM	Wean	133	91	103	81	28
	Mid-test	128	133	123	117	38
	Off-test	168	184	204	194	61
LOIN	Wean	112	83	92	95	23
	Mid-test	91	141	188	105	29
	Off-test	81	206	112	92	74
SCOL	Wean	73	60	61	76	19
	Mid-test	69	69	76	69	6
	Off-test	126	73	121	119	30
PH	Wean	88	77	83	86	18
	Mid-test	119	92	84	101	29
	Off-test	94	85	92	109	21
IMF	Wean	108	101	117	81	26
	Mid-test	320	292	227	142	71
	Off-test	168	188	151	147	53
MINA	Wean	60	68	76	53	17
	Mid-test	87	91	82	90	24
	Off-test	186	125	171	209	48
MINB	Wean	64	52	72	78	16
	Mid-test	94	94	74	101	25
	Off-test	121	93	94	121	35
MINL	Wean	88	61	59	53	17
	Mid-test	70	108	65	108	21
	Off-test	115	78	71	95	19
SFIRM	Wean	61	56	49	51	20
	Mid-test	278	206	114	280	45
	Off-test	184	143	121	156	38

SMARB	Wean	96	116	82	92	24
	Mid-test	114	185	166	142	38
	Off-test	98	101	112	104	30
SSF	Wean	97	69	86	91	23
	Mid-test	102	80	89	198	20
	Off-test	263	93	123	116	27

Table S8. Number of significant markers of each fold of cross-validation and the number of common markers for each trait at each stage within each fold

Traits[1]	Fold 1	Fold 2	Fold 3	Fold 4	Common
LD	2926	2492	2944	2739	477
FD	4610	4189	5091	4432	1101
CADG	3236	2944	3535	3560	598
BEL	3458	3158	3950	3861	722
HAM	2929	2905	3035	2917	477
LOIN	3709	2978	3099	3384	591
SCOL	3376	3504	2981	2978	561
PH	2466	2123	2425	2377	593
IMF	3660	3993	3887	3542	797
MINA	2917	3158	3240	2679	501
MINB	2533	2540	2679	2605	539
MINL	3322	3403	2889	3323	405
SFIRM	2960	3009	2618	2889	487
SMARB	3448	3577	3916	3207	586
SSF	3046	3085	3138	3008	481

[1]LD = Loin depth; FD = Fat depth; CADG = Carcass average daily gain; HAM = Ham weight; LOIN = Loin weight; BEL = Belly weight; IMF = Intramuscular fat percent, MINA = Minolta a*, MINB = Minolta b*, MINL = Minolta L*, PH = Ultimate pH; SCOL = Subjective color score; SMARB = Subjective marbling score; SFIRM = Subjective firmness score; SSF = Slice shear force
