# Supplementary material for: Modeling host-microbiome interactions for the prediction of meat quality and carcass composition traits in swine
Source: Genet Sel Evol. 2020 Jul 29;52:41. doi: 10.1186/s12711-020-00561-7 (PMC7388461; doi:10.1186/s12711-020-00561-7)
Supplement: Supplementary file 5 — Additional file 5: Table S9. Variance components explained by microbiome relationship matrix ($\bf O$O), genomic relationship matrix ($\bf G$G), pen ($\bf P$P), residual ($\bf R$R), microbiability (m2) and heritability (h2) in different models. Table S10. Variance components explained by microbiome relationship matrix ($\bf O$O), pen ($\bf P$P), residual ( [file 12711_2020_561_MOESM5_ESM.pdf]

Table S9. Variance components explained by microbiome relationship matrix (O), genomic relationship matrix (G), pen (P), residual (R), microbiability (m^2) and heritability (h^2) in different models[1]

Traits[2]	Effects	M1	Wean	Mid-test	Off-test
LD	P	6.55±1.67	6.55±1.67	6.42±1.67	6.49±1.66
	O	-	0.29E-04±0.00	1.76±1.58	0.71±1.05
	G	6.15±2.94	6.15±2.93	5.88±2.36	5.88±2.36
	R	38.53±2.06	38.53±2.06	37.20±2.15	38.09±2.05
	m^2	-	0.00±0.00	0.03+0.03	0.01±0.02
	h^2	0.12±0.04	0.11±0.04	0.12±0.04	0.11±0.05
FD	P	2.78±0.62	2.37±0.60	2.90±0.67	2.77±0.65
	O	-	5.37±1.05	2.71±0.87	0.18±0.48
	G	9.84±1.55	7.61±1.34	9.00±1.48	9.80±1.56
	R	9.99±1.05	6.56±1.05	7.83±1.13	9.84±1.14
	m^2	-	0.25±0.04	0.12+0.03	0.01±0.02
	h^2	0.44±0.05	0.34±0.05	0.40±0.05	0.43±0.05
CADG	P	0.78E-05±0.00	0.59E-05±0.00	0.61E-05 ± 0.00	0.72E-0±0.00
	O	-	1.18±0.29	0.95±0.26	0.31±0.18
	G	1.05±0.23	0.94±0.27	1.07±0.29	0.98±0.08
	R	4.71±0.29	3.18±0.30	3.31±0.26	4.63±0.30
	m^2	-	0.22±0.05	0.18+0.04	0.06±0.03
	h^2	0.20±0.05	0.18±0.04	0.20±0.05	0.18±0.05
HAM	P	0.87E-07±00	0.73E-05±0.0	0.72E-05±0.00	0.85E-05±0.00
	O	-	0.83±0.29	0.80±0.25	0.10±0.16
	G	0.72±0.27	0.73±0.27	0.82±0.28	0.70±0.27
	R	4.71±0.29	3.96±0.33	3.96±0.33	4.65±0.32
	m^2	-	0.15±0.05	0.14+0.04	0.02±0.02
	h^2	0.13±0.05	0.13±0.04	0.13±0.05	0.13±0.04
LOIN	P	0.82E-05±0.0	0.70E-05±00	0.70E-05±00	0.80E-05±0.00
	O	-	0.44±0.18	0.43±0.16	0.10±0.16
	G	0.62±0.18	0.64±0.18	0.65±0.18	0.58±0.17
	R	2.87±0.18	2.45±0.21	2.46±0.21	2.79±0.19
	m^2	-	0.13±0.05	0.12+0.04	0.03±0.02
	h^2	0.18±0.05	0.18±0.04	0.18±0.05	0.17±0.05
BEL	P	0.79E-05±0.0	0.54E-05±0.00	0.59E-05±0.00	0.76E-05±0.00
	O	-	1.89±0.38	1.37±0.38	0.28±0.20
	G	1.34±0.36	1.18±0.33	1.39±0.36	1.28±0.35
	R	5.09±0.34	3.50±0.37	3.45±0.38	4.88±0.36
	m^2	-	0.29±0.05	0.20+0.04	0.04±0.03

	h^2	0.21±0.05	0.18±0.04	0.21±0.05	0.19±0.05
IMF	P	0.04 ± 0.02	0.04 ± 0.02	0.04± 0.02	0.04±0.02
	O	-	0.06±0.03	0.03±0.02	0.03±0.02
	G	0.55±0.08	0.53±0.08	0.54±0.08	0.55±0.08
	R	0.41±0.05	0.37±0.37	0.39±0.05	0.38±0.04
	m^2	-	0.06±0.02	0.03+0.02	0.03±0.02
	h^2	0.55±0.05	0.53±0.05	0.54±0.05	0.54±0.05
SMARB	P	0.08 ± 0.02	0.08 ± 0.02	0.08 ± 0.02	0.08 ± 0.02
	O	-	0.007±0.02	0.06±0.02	0.02±0.03
	G	0.26±0.08	0.26±0.06	0.26±0.06	0.26±0.06
	R	0.48±0.04	0.47±0.05	0.43±0.05	0.46±0.04
	m^2	-	0.01±0.02	0.07+0.02	0.02±0.02
	h^2	0.32±0.05	0.32±0.05	0.31±0.05	0.32±0.05
MINA	P	0.19 ± 0.04	0.17 ± 0.03	0.18 ± 0.03	0.18 ± 0.03
	O	-	0.12±0.05	0.02±0.04	0.23E-04±0.00
	G	0.22±0.06	0.22±0.06	0.22±0.06	0.22±0.06
	R	0.85±0.06	0.77±0.07	0.83±0.05	0.85±0.06
	m^2	-	0.09±0.02	0.02+0.02	0.00±0.00
	h^2	0.17±0.05	0.16±0.05	0.17±0.05	0.17±0.05
MINB	P	0.15 ± 0.03	0.13 ± 0.03	0.14 ± 0.03	0.15 ± 0.03
	O	-	0.08±0.03	0.005±0.01	0.64E-05±0.00
	G	0.056±0.03	0.056±0.03	0.058±0.03	0.056±0.03
	R	0.49±0.03	0.42±0.04	0.48±0.04	0.49±0.04
	m^2	-	0.11±0.04	0.007+0.02	0.00±0.00
	h^2	0.08±0.04	0.08±0.04	0.08±0.04	0.08±0.04
MINL	P	6.15 ± 1.16	6.15 ± 1.16	6.15 ± 1.16	6.15 ± 1.16
	O	-	1.16±1.15	0.99E-05±0.00	0.61E-05±0.00
	G	6.90±1.82	6.57±1.78	6.91±1.82	6.91±1.82
	R	20.05±1.63	19.23±1.81	20.04±1.52	20.04±1.52
	m^2	-	0.03±0.03	0.00+0.00	0.00±0.00
	h^2	0.21±0.04	0.19±0.05	0.21±0.05	0.21±0.05
PH	P	0.013 ± 0.002	0.013 ± 0.002	0.013 ± 0.002	0.013 ± 0.002
	O	-	0.002±0.002	0.001±0.007	0.26E-05±0.00
	G	0.003±0.001	0.003±0.001	0.003±0.001	0.003±0.001
	R	0.031±0.002	0.031±0.002	0.031±0.002	0.031±0.002
	m^2	-	0.04±0.03	0.002+0.01	0.00±0.00
	h^2	0.06±0.04	0.06±0.04	0.06±0.04	0.06±0.04
SCOL	P	0.014 ± 0.006	0.013 ± 0.006	0.014 ± 0.006	0.013 ± 0.006
	O	-	0.012±0.011	0.40E-05±0.00	0.41E-05±0.00
	G	0.096±0.02	0.097±0.02	0.096±0.02	0.096±0.02

	R	0.216±0.018	0.204±0.019	0.215±0.018	0.215±0.018
	m^2	-	0.04±0.03	0.002+0.01	0.00±0.00
	h^2	0.30±0.06	0.30±0.06	0.30±0.06	0.30±0.05
SFIRM	P	0.026 ± 0.029	0.022 ± 0.028	0.028 ± 0.029	0.026 ± 0.029
	O	-	0.012±0.011	0.40E-05±0.00	0.41E-05±0.00
	G	0.134±0.050	0.122±0.046	0.120±0.044	0.134±0.050
	R	0.904±0.059	0.791±0.068	0.834±0.063	0.904±0.059
	m^2	-	0.13±0.04	0.08+0.03	0.00±0.00
	h^2	0.13±0.04	0.11±0.04	0.11±0.04	0.13±0.05
SSF	P	0.52±0.36	0.49±0.36	0.50±0.36	0.52±0.36
	O	-	0.30±0.36	1.38±0.59	0.41±0.32
	G	3.07±0.74	3.03±0.73	3.00±0.73	3.12±0.75
	R	9.42±0.70	9.20±0.66	8.27±0.78	8.99±0.77
	m^2	-	0.02±0.02	0.10+0.04	0.03±0.02
	h^2	0.24±0.05	0.23±0.05	0.22±0.05	0.24±0.05

[1]M1 contains **G** matrix and pen effect as random effect. Models at Wean, On-test and Off-test contains microbiome information at weaning, on-test and off-test respectively.

[2]LD = Loin depth; FD = Fat depth; CADG = Carcass average daily gain; IMF = Intramuscular fat percent, MINA = Minolta a*, MINB = Minolta b*, MINL = Minolta L*, PH = Ultimate pH; SCOL = Subjective color score; SMARB = Subjective marbling score; SFIRM = Subjective firmness score; SSF = Slice shear force, HAM = Ham weight; LOIN = Loin weight; BEL = Belly weight.

Table S10: Variance components explained by microbiome relationship matrix (O), pen (P), residual (R) and microbiability (m^2) at different stages of production when only microbiome information was included in the model

Traits[2]	Effects	Wean	Mid-test	Off-test
LD	P	7.35±1.71	7.30±1.71	7.47±1.71
	O	1.16±1.19	2.27±1.69	2.3E-05±0.00
	R	42.52±2.35	41.62±2.48	43.56±2.17
	m^2	0.03±0.02	0.04±0.03	0.00±0.00
FD	P	4.33±0.79	4.49±0.78	3.76±0.71
	O	0.38±0.57	3.64±1.03	6.96±1.24
	R	17.59±1.01	14.29±1.01	11.54±0.97
	m^2	0.02±0.02	0.16±0.04	0.31±0.05
CADG	P	4.86E-06±0.00	4.86E-07±0.00	4.86E-07±0.00
	O	0.36±0.19	0.97±0.27	1.20±0.3
	R	4.80±0.25	4.27±0.26	4.03±0.27
	m^2	0.07±0.03	0.19±0.04	0.23±0.09
HAM	P	2.09E-08±0.00	2.63E-07±0.00	2.57E-07±0.00
	O	0.08±0.01	0.41±0.27	0.50±0.02
	R	2.89±0.02	2.60±0.26	2.52±0.02
	m^2	0.03±0.03	0.13±0.04	0.16±0.05
LOIN	P	4.24E-06±0.00	3.07E-07±0.00	3.03E-07±0.00
	O	0.19±0.12	0.41±0.16	0.43±0.02
	R	3.27±0.17	3.07±0.18	3.06±0.18
	m^2	0.06±0.03	0.12±0.04	0.14±0.05
BEL	P	4.71E-06±0.00	3.95E-07±0.00	3.64E-07±0.00
	O	0.29±0.12	1.18±0.35	1.47±0.39
	R	4.69±0.17	3.95±0.31	3.56±0.32
	m^2	0.06±0.03	0.22±0.04	0.29±0.05
IMF	P	0.12±0.03	0.10±0.03	0.11±0.03
	O	0.04±0.03	0.05±0.03	0.10±0.04
	R	0.80±0.05	0.79±0.05	0.74±0.05
	m^2	0.04±0.03	0.05±0.03	0.11±0.04
SMARB	P	0.11±0.03	0.10±0.03	0.11±0.03
	O	6E-08±0.00	0.05±0.03	0.02±0.02
	R	0.69±0.03	0.64±0.04	0.67±0.04

	m^2	0.00±0.00	0.07±0.03	0.03±0.02
MINA	P	0.25±0.05	0.24±0.04	0.22±0.03
	O	9E-08±0.00	0.03±0.03	0.15±0.06
	R	1.12±0.05	0.99±0.06	0.90±0.06
	m^2	0.00±0.00	0.03±0.03	0.12±0.04
MINB	P	0.14±0.03	0.15±0.02	0.14±0.03
	O	3.8E-06±0.00	0.007±0.01	0.08±0.03
	R	0.54±0.05	0.53±0.03	0.47±0.03
	m^2	0.00±0.00	0.01±0.02	0.11±0.04
MINL	P	6.53±1.12	6.93±1.23	6.66±1.21
	O	2.4E-06±0.00	0.18±0.67	2.70±1.45
	R	23.4±1.17	25.76±1.42	23.66±1.63
	m^2	0.00±0.00	0.005±0.02	0.08±0.04
PH	P	0.012±0.002	0.013±0.002	0.013±0.002
	O	1.5E-09±0.00	0.00016±0.0007	0.002±0.001
	R	0.033±0.001	0.033±0.002	0.031±0.002
	m^2	0.00±0.00	0.003±0.01	0.04±0.03
SCOL	P	0.03±0.01	0.03±0.01	0.03±0.00
	O	1.2E-09±0.00	1.8E-07±0.00	0.03±0.01
	R	0.29±0.01	0.29±0.01	0.280.01
	m^2	0.00±0.00	0.00±0.00	0.06±0.04
SFIRM	P	0.05±0.03	0.05±0.03	0.04±0.03
	O	4.2E-07±0.00	5.8E-07±0.04	0.14±0.05
	R	1.00±0.05	0.97±0.05	0.88±0.05
	m^2	0.00±0.00	0.00±0.00	0.14±0.04
SSF	P	1.34±0.42	1.28±0.41	1.29±0.42
	O	0.23±0.36	1.63±0.64	0.38±0.05
	R	11.55±0.68	10.40±0.71	11.43±0.67
	m^2	0.01±0.02	0.12±0.05	0.03±0.03

[2]LD = Loin depth; FD = Fat depth; CADG = Carcass average daily gain; IMF = Intramuscular fat percent, MINA = Minolta a*, MINB = Minolta b*, MINL = Minolta L*, PH = Ultimate pH; SCOL = Subjective color score; SMARB = Subjective marbling score; SFIRM = Subjective firmness score; SSF = Slice shear force, HAM = Ham weight; LOIN = Loin weight; BEL = Belly weight
