# Supplementary material for: Modeling host-microbiome interactions for the prediction of meat quality and carcass composition traits in swine
Source: Genet Sel Evol. 2020 Jul 29;52:41. doi: 10.1186/s12711-020-00561-7 (PMC7388461; doi:10.1186/s12711-020-00561-7)
Supplement: Supplementary file 6 — Additional file 6: Table S11. Predictive ability of Loin depth for each fold at different complexities and stages and with different models. Table S12. Predictive ability of fat depth for each fold at different complexities and stages and with different models. Table S13. Predictive ability of carcass average daily gain for each fold at different complexities and stages and with different models. Table S14. Predictive ability of ham weight for each fold at different complexities and stages and with different models. Table S15. Predictive ability of loin weight for each fold at different complexities and stages and with different models. Table S16. Predictive ability of belly weight for each fold at different complexities and stages and with different models. Table S17. Predictive ability of intramuscular fat (%) for each fold at different complexities and stages and with different models. Table S18. Predictive ability of Minolta a* for each fold at different complexities and stages and with different models. Table S19. Predictive ability of Minolta b* for each fold at different complexities and stages and with different models. Table S20. Predictive ability of Minolta L* for each fold at different complexities and stages and with different models. Table S21. Predictive ability of pH for each fold at different complexities and stages and with different models. Table S22. Predictive ability of subjective color score for each fold at different complexities and stages and with different models. Table S23. Predictive ability of subjective marbling score for each fold at different complexities and stages and with different models. Table S24. Predictive ability of Subjective firmness score for each fold at different complexities and stages and with different models. Table S25. Predictive ability of slice shear force for each fold at different complexities and stages and with different models. [file 12711_2020_561_MOESM6_ESM.pdf]

Table S11: Predictive ability of Loin depth for each fold at different complexity, stage and model.

Complexity	Stage	Model[1]	Fold1	Fold2	Fold3	Fold4	Average	SD
Full	Wean	M1	0.17	0.06	0.14	0.21	0.15	0.06
Full	Mid-test	M1	0.17	0.06	0.14	0.21	0.15	0.06
Full	Off-test	M1	0.17	0.06	0.14	0.21	0.15	0.06
Full	Wean	M2	0.15	0.08	0.12	0.16	0.13	0.04
Full	Mid-test	M2	0.14	0.04	0.09	0.17	0.11	0.06
Full	Off-test	M2	0.1	0.02	0.06	0.14	0.08	0.05
Full	Wean	M3	0.18	0.10	0.13	0.20	0.15	0.05
Full	Mid-test	M3	0.17	0.07	0.12	0.21	0.14	0.06
Full	Off-test	M3	0.15	0.05	0.10	0.20	0.12	0.06
Full	Wean	M4	0.18	0.09	0.13	0.20	0.15	0.05
Full	Mid-test	M4	0.17	0.07	0.12	0.21	0.14	0.06
Full	Off-test	M4	0.14	0.05	0.1	0.19	0.12	0.06
Informatively reduced	Wean	M1	0.16	0.11	0.15	0.32	0.19	0.13
Informatively reduced	Mid-test	M1	0.16	0.11	0.15	0.32	0.19	0.13
Informatively reduced	Off-test	M1	0.16	0.11	0.15	0.32	0.19	0.13
Informatively reduced	Wean	M2	0.12	0.29	0.06	0.29	0.19	0.12
Informatively reduced	Mid-test	M2	0.07	0.32	0.00	0.27	0.17	0.15
Informatively reduced	Off-test	M2	0.07	0.29	0.07	0.30	0.18	0.16
Informatively reduced	Wean	M3	0.10	0.08	0.10	0.13	0.10	0.02
Informatively reduced	Mid-test	M3	0.09	0.09	0.07	0.13	0.10	0.03
Informatively reduced	Off-test	M3	0.07	0.07	0.10	0.11	0.09	0.02
Informatively reduced	Wean	M4	0.10	0.06	0.10	0.14	0.10	0.03
Informatively reduced	Mid-test	M4	0.09	0.07	0.07	0.14	0.09	0.03
Informatively reduced	Off-test	M4	0.07	0.06	0.09	0.11	0.08	0.02
Randomly reduced	Wean	M1	0.18	0.07	0.13	0.21	0.15	0.04
Randomly reduced	Mid-test	M1	0.18	0.07	0.13	0.21	0.15	0.04
Randomly reduced	Off-test	M1	0.18	0.07	0.13	0.21	0.15	0.04
Randomly reduced	Wean	M2	0.14	0.06	0.11	0.16	0.12	0.03
Randomly reduced	Mid-test	M2	0.13	0.04	0.11	0.15	0.11	0.04
Randomly reduced	Off-test	M2	0.10	0.04	0.09	0.14	0.09	0.03
Randomly reduced	Wean	M3	0.19	0.08	0.13	0.21	0.15	0.04
Randomly reduced	Mid-test	M3	0.17	0.08	0.13	0.21	0.15	0.04
Randomly reduced	Off-test	M3	0.16	0.07	0.12	0.20	0.14	0.04
Randomly reduced	Wean	M4	0.19	0.08	0.12	0.22	0.15	0.04
Randomly reduced	Mid-test	M4	0.17	0.07	0.13	0.21	0.15	0.04
Randomly reduced	Off-test	M4	0.16	0.06	0.12	0.19	0.13	0.03

Table S12: Predictive ability of Fat depth for each fold at different complexity, stage and model.

Complexity	Stage	Model	Fold1	Fold2	Fold3	Fold4	Average	SD
Full	Wean	M1	0.55	0.51	0.47	0.57	0.53	0.06
Full	Mid-test	M1	0.55	0.51	0.47	0.57	0.53	0.06
Full	Off-test	M1	0.55	0.51	0.47	0.57	0.53	0.06
Full	Wean	M2	0.46	0.42	0.37	0.50	0.44	0.06
Full	Mid-test	M2	0.56	0.44	0.41	0.55	0.49	0.08
Full	Off-test	M2	0.58	0.52	0.48	0.51	0.52	0.04
Full	Wean	M3	0.61	0.52	0.43	0.57	0.52	0.06
Full	Mid-test	M3	0.61	0.54	0.46	0.60	0.56	0.07
Full	Off-test	M3	0.63	0.58	0.5	0.58	0.57	0.05
Full	Wean	M4	0.61	0.52	0.43	0.57	0.52	0.06
Full	Mid-test	M4	0.61	0.54	0.46	0.61	0.56	0.07
Full	Off-test	M4	0.61	0.58	0.49	0.61	0.584	0.06
Informatively reduced	Wean	M1	0.61	0.56	0.44	0.64	0.55	0.08
Informatively reduced	Mid-test	M1	0.61	0.56	0.44	0.64	0.55	0.08
Informatively reduced	Off-test	M1	0.61	0.56	0.44	0.64	0.55	0.08
Informatively reduced	Wean	M2	0.52	0.5	0.39	0.46	0.47	0.06
Informatively reduced	Mid-test	M2	0.58	0.52	0.42	0.48	0.5	0.06
Informatively reduced	Off-test	M2	0.61	0.59	0.51	0.59	0.55	0.04
Informatively reduced	Wean	M3	0.46	0.53	0.41	0.51	0.48	0.05
Informatively reduced	Mid-test	M3	0.52	0.55	0.44	0.56	0.52	0.05
Informatively reduced	Off-test	M3	0.54	0.58	0.45	0.55	0.53	0.06
Informatively reduced	Wean	M4	0.47	0.52	0.41	0.52	0.48	0.05
Informatively reduced	Mid-test	M4	0.52	0.55	0.43	0.56	0.52	0.06
Informatively reduced	Off-test	M4	0.55	0.58	0.45	0.56	0.54	0.06
Randomly reduced	Wean	M1	0.55	0.50	0.42	0.54	0.50	0.07
Randomly reduced	Mid-test	M1	0.55	0.50	0.42	0.54	0.50	0.07
Randomly reduced	Off-test	M1	0.55	0.50	0.42	0.54	0.50	0.07
Randomly reduced	Wean	M2	0.48	0.41	0.36	0.50	0.44	0.05
Randomly reduced	Mid-test	M2	0.54	0.43	0.39	0.53	0.45	0.06
Randomly reduced	Off-test	M2	0.53	0.45	0.42	0.50	0.48	0.06
Randomly reduced	Wean	M3	0.55	0.51	0.42	0.55	0.51	0.06
Randomly reduced	Mid-test	M3	0.58	0.52	0.45	0.57	0.53	0.07
Randomly reduced	Off-test	M3	0.57	0.53	0.46	0.56	0.53	0.06
Randomly reduced	Wean	M4	0.55	0.51	0.42	0.55	0.51	0.06
Randomly reduced	Mid-test	M4	0.58	0.52	0.45	0.57	0.51	0.07
Randomly reduced	Off-test	M4	0.56	0.53	0.45	0.56	0.53	0.06

Table S13: Predictive ability of Carcass average daily gain for each fold at different complexity, stage and model.

Complexity	Stage	Model	Fold1	Fold2	Fold3	Fold4	Average	SD
Full	Wean	M1	0.26	0.27	0.24	0.25	0.26	0.01
Full	Off-test	M1	0.26	0.27	0.24	0.25	0.26	0.01
Full	Mid-test	M1	0.26	0.27	0.24	0.25	0.26	0.01
Full	Wean	M2	0.27	0.24	0.24	0.28	0.26	0.02
Full	Mid-test	M2	0.35	0.22	0.28	0.32	0.29	0.06
Full	Off-test	M2	0.37	0.31	0.3	0.31	0.32	0.03
Full	Wean	M3	0.28	0.29	0.26	0.29	0.28	0.01
Full	Mid-test	M3	0.34	0.28	0.3	0.32	0.31	0.03
Full	Off-test	M3	0.36	0.35	0.3	0.33	0.34	0.03
Full	Wean	M4	0.28	0.29	0.26	0.29	0.28	0.01
Full	Mid-test	M4	0.34	0.28	0.29	0.32	0.31	0.03
Full	Off-test	M4	0.35	0.34	0.3	0.33	0.33	0.02
Informatively reduced	Wean	M1	0.21	0.29	0.22	0.52	0.31	0.14
Informatively reduced	Mid-test	M1	0.21	0.29	0.22	0.52	0.31	0.14
Informatively reduced	Off-test	M1	0.21	0.29	0.22	0.52	0.31	0.14
Informatively reduced	Wean	M2	0.20	0.40	0.18	0.41	0.3	0.12
Informatively reduced	Mid-test	M2	0.28	0.49	0.3	0.53	0.4	0.13
Informatively reduced	Off-test	M2	0.34	0.42	0.36	0.45	0.39	0.05
Informatively reduced	Wean	M3	0.23	0.35	0.21	0.29	0.27	0.06
Informatively reduced	Mid-test	M3	0.29	0.43	0.27	0.34	0.33	0.07
Informatively reduced	Off-test	M3	0.28	0.39	0.28	0.31	0.32	0.05
Informatively reduced	Wean	M4	0.23	0.34	0.21	0.29	0.27	0.06
Informatively reduced	Mid-test	M4	0.29	0.43	0.27	0.34	0.33	0.07
Informatively reduced	Off-test	M4	0.28	0.39	0.28	0.30	0.31	0.05
Randomly reduced	Wean	M1	0.27	0.27	0.24	0.24	0.26	0.02
Randomly reduced	Mid-test	M1	0.27	0.27	0.24	0.24	0.26	0.02
Randomly reduced	Off-test	M1	0.27	0.27	0.24	0.24	0.26	0.02
Randomly reduced	Wean	M2	0.27	0.22	0.23	0.25	0.24	0.02
Randomly reduced	Mid-test	M2	0.29	0.21	0.23	0.25	0.25	0.03
Randomly reduced	Off-test	M2	0.29	0.21	0.23	0.24	0.24	0.03
Randomly reduced	Wean	M3	0.28	0.27	0.25	0.26	0.27	0.01
Randomly reduced	Mid-test	M3	0.29	0.27	0.26	0.26	0.27	0.01
Randomly reduced	Off-test	M3	0.29	0.27	0.25	0.26	0.27	0.02
Randomly reduced	Wean	M4	0.28	0.27	0.25	0.26	0.27	0.02
Randomly reduced	Mid-test	M4	0.29	0.26	0.26	0.26	0.27	0.01
Randomly reduced	Off-test	M4	0.28	0.27	0.25	0.26	0.27	0.02

Table S14: Predictive ability of Ham weight for each fold at different complexity, stage and model.

Complexity	Stage	Model	Fold1	Fold2	Fold3	Fold4	Average	SD
Full	Wean	M1	0.13	0.13	0.1	0.12	0.12	0.01
Full	Mid-test	M1	0.13	0.13	0.1	0.12	0.12	0.01
Full	Off-test	M1	0.13	0.13	0.1	0.12	0.12	0.01
Full	Wean	M2	0.09	0.10	0.10	0.19	0.12	0.05
Full	Mid-test	M2	0.18	0.09	0.14	0.22	0.16	0.06
Full	Off-test	M2	0.19	0.19	0.13	0.23	0.18	0.04
Full	Wean	M3	0.13	0.15	0.1	0.16	0.14	0.03
Full	Mid-test	M3	0.20	0.14	0.16	0.21	0.18	0.03
Full	Off-test	M3	0.21	0.22	0.13	0.23	0.20	0.05
Full	Wean	M4	0.12	0.15	0.11	0.17	0.14	0.03
Full	Mid-test	M4	0.19	0.14	0.15	0.20	0.17	0.03
Full	Off-test	M4	0.20	0.21	0.14	0.22	0.19	0.04
Informatively reduced	Wean	M1	0.16	0.18	0.09	0.58	0.25	0.22
Informatively reduced	Mid-test	M1	0.16	0.18	0.09	0.58	0.25	0.22
Informatively reduced	Off-test	M1	0.16	0.18	0.09	0.58	0.25	0.22
Informatively reduced	Wean	M2	0.02	0.27	0.04	0.38	0.18	0.18
Informatively reduced	Mid-test	M2	0.09	0.37	0.14	0.38	0.24	0.15
Informatively reduced	Off-test	M2	0.16	0.35	0.19	0.3	0.25	0.09
Informatively reduced	Wean	M3	0.16	0.22	0.1	0.17	0.16	0.05
Informatively reduced	Mid-test	M3	0.17	0.33	0.13	0.17	0.2	0.09
Informatively reduced	Off-test	M3	0.20	0.27	0.14	0.19	0.2	0.05
Informatively reduced	Wean	M4	0.16	0.21	0.10	0.16	0.16	0.04
Informatively reduced	Mid-test	M4	0.18	0.31	0.13	0.17	0.2	0.08
Informatively reduced	Off-test	M4	0.20	0.27	0.14	0.19	0.2	0.05
Randomly reduced	Wean	M1	0.14	0.12	0.1	0.12	0.12	0.02
Randomly reduced	Mid-test	M1	0.14	0.12	0.1	0.12	0.12	0.02
Randomly reduced	Off-test	M1	0.14	0.12	0.1	0.12	0.12	0.02
Randomly reduced	Wean	M2	0.1	0.08	0.09	0.15	0.11	0.00
Randomly reduced	Mid-test	M2	0.12	0.08	0.09	0.16	0.11	0.01
Randomly reduced	Off-test	M2	0.12	0.08	0.08	0.15	0.11	0.02
Randomly reduced	Wean	M3	0.14	0.12	0.09	0.14	0.12	0.02
Randomly reduced	Mid-test	M3	0.16	0.12	0.1	0.15	0.13	0.02
Randomly reduced	Off-test	M3	0.17	0.12	0.09	0.15	0.13	0.03
Randomly reduced	Wean	M4	0.14	0.12	0.09	0.13	0.12	0.02
Randomly reduced	Mid-test	M4	0.16	0.13	0.1	0.14	0.13	0.02
Randomly reduced	Off-test	M4	0.16	0.13	0.1	0.15	0.14	0.16

Table S15: Predictive ability of Loin weight for each fold at different complexity, stage and model.

Complexity	Stage	Model	Fold1	Fold2	Fold3	Fold4	Average	SD
Full	Wean	M1	0.16	0.16	0.15	0.14	0.15	0.01
Full	Mid-test	M1	0.16	0.16	0.15	0.14	0.15	0.01
Full	Off-test	M1	0.16	0.16	0.15	0.14	0.15	0.01
Full	Wean	M2	0.19	0.14	0.14	0.19	0.15	0.03
Full	Mid-test	M2	0.24	0.07	0.15	0.22	0.17	0.08
Full	Off-test	M2	0.20	0.17	0.16	0.21	0.18	0.02
Full	Wean	M3	0.18	0.18	0.16	0.17	0.17	0.01
Full	Mid-test	M3	0.23	0.13	0.19	0.19	0.18	0.04
Full	Off-test	M3	0.20	0.22	0.20	0.20	0.20	0.01
Full	Wean	M4	0.18	0.18	0.16	0.17	0.17	0.01
Full	Mid-test	M4	0.23	0.14	0.19	0.19	0.19	0.04
Full	Off-test	M4	0.20	0.21	0.20	0.19	0.20	0.01
Informatively reduced	Wean	M1	0.11	0.16	0.12	0.60	0.25	0.24
Informatively reduced	Mid-test	M1	0.11	0.16	0.12	0.6	0.25	0.24
Informatively reduced	Off-test	M1	0.11	0.16	0.12	0.6	0.25	0.24
Informatively reduced	Wean	M2	0.10	0.27	0.08	0.40	0.21	0.15
Informatively reduced	Mid-test	M2	0.23	0.46	0.15	0.50	0.34	0.17
Informatively reduced	Off-test	M2	0.17	0.32	0.2	0.34	0.26	0.08
Informatively reduced	Wean	M3	0.13	0.21	0.14	0.11	0.15	0.04
Informatively reduced	Mid-test	M3	0.25	0.33	0.17	0.13	0.22	0.09
Informatively reduced	Off-test	M3	0.15	0.24	0.18	0.14	0.18	0.04
Informatively reduced	Wean	M4	0.13	0.19	0.14	0.12	0.15	0.03
Informatively reduced	Mid-test	M4	0.25	0.31	0.18	0.12	0.22	0.08
Informatively reduced	Off-test	M4	0.15	0.24	0.18	0.14	0.18	0.04
Randomly reduced	Wean	M1	0.19	0.17	0.13	0.11	0.15	0.02
Randomly reduced	Mid-test	M1	0.19	0.17	0.13	0.11	0.15	0.02
Randomly reduced	Off-test	M1	0.19	0.17	0.13	0.11	0.15	0.02
Randomly reduced	Wean	M2	0.18	0.11	0.13	0.18	0.15	0.02
Randomly reduced	Mid-test	M2	0.17	0.08	0.1	0.15	0.13	0.04
Randomly reduced	Off-test	M2	0.16	0.1	0.09	0.15	0.13	0.04
Randomly reduced	Wean	M3	0.2	0.17	0.15	0.14	0.17	0.01
Randomly reduced	Mid-test	M3	0.19	0.15	0.13	0.12	0.15	0.02
Randomly reduced	Off-test	M3	0.19	0.17	0.13	0.12	0.15	0.02
Randomly reduced	Wean	M4	0.19	0.17	0.15	0.13	0.16	0.01
Randomly reduced	Mid-test	M4	0.19	0.15	0.13	0.12	0.15	0.02
Randomly reduced	Off-test	M4	0.19	0.17	0.13	0.12	0.15	0.02

Table S16: Predictive ability of Belly weight for each fold at different complexity, stage and model.

Complexity	Stage	Model	Fold1	Fold2	Fold3	Fold4	Average	SD
Full	Wean	M1	0.23	0.31	0.16	0.18	0.22	0.07
Full	Mid-test	M1	0.23	0.31	0.16	0.18	0.22	0.07
Full	Off-test	M1	0.23	0.31	0.16	0.18	0.22	0.07
Full	Wean	M2	0.19	0.21	0.19	0.18	0.19	0.01
Full	Mid-test	M2	0.33	0.2	0.22	0.28	0.26	0.06
Full	Off-test	M2	0.35	0.31	0.30	0.22	0.30	0.05
Full	Wean	M3	0.24	0.31	0.18	0.22	0.24	0.05
Full	Mid-test	M3	0.36	0.30	0.23	0.30	0.30	0.05
Full	Off-test	M3	0.37	0.38	0.27	0.26	0.30	0.06
Full	Wean	M4	0.24	0.31	0.19	0.22	0.24	0.05
Full	Mid-test	M4	0.36	0.3	0.23	0.29	0.30	0.05
Full	Off-test	M4	0.37	0.37	0.27	0.26	0.32	0.06
Informatively reduced	Wean	M1	0.22	0.35	0.18	0.53	0.32	0.16
Informatively reduced	Mid-test	M1	0.22	0.35	0.18	0.53	0.32	0.16
Informatively reduced	Off-test	M1	0.22	0.35	0.18	0.53	0.32	0.16
Informatively reduced	Wean	M2	0.18	0.43	0.17	0.41	0.30	0.14
Informatively reduced	Mid-test	M2	0.39	0.47	0.25	0.47	0.40	0.10
Informatively reduced	Off-test	M2	0.36	0.49	0.35	0.44	0.41	0.07
Informatively reduced	Wean	M3	0.24	0.41	0.17	0.25	0.27	0.10
Informatively reduced	Mid-test	M3	0.36	0.5	0.23	0.35	0.36	0.11
Informatively reduced	Off-test	M3	0.34	0.49	0.22	0.32	0.34	0.11
Informatively reduced	Wean	M4	0.24	0.41	0.17	0.25	0.27	0.10
Informatively reduced	Mid-test	M4	0.35	0.51	0.23	0.35	0.37	0.11
Informatively reduced	Off-test	M4	0.33	0.49	0.22	0.32	0.35	0.11
Randomly reduced	Mid-test	M1	0.23	0.31	0.15	0.17	0.22	0.08
Randomly reduced	Off-test	M1	0.23	0.31	0.15	0.17	0.22	0.08
Randomly reduced	Wean	M1	0.23	0.31	0.15	0.17	0.22	0.08
Randomly reduced	Wean	M2	0.17	0.19	0.18	0.14	0.17	0.02
Randomly reduced	Mid-test	M2	0.19	0.16	0.14	0.17	0.17	0.03
Randomly reduced	Off-test	M2	0.22	0.18	0.19	0.14	0.18	0.02
Randomly reduced	Wean	M3	0.24	0.32	0.16	0.19	0.23	0.07
Randomly reduced	Mid-test	M3	0.25	0.30	0.17	0.20	0.23	0.07
Randomly reduced	Off-test	M3	0.27	0.30	0.18	0.20	0.24	0.06
Randomly reduced	Wean	M4	0.24	0.31	0.16	0.18	0.22	0.06
Randomly reduced	Mid-test	M4	0.25	0.29	0.17	0.19	0.23	0.06
Randomly reduced	Off-test	M4	0.26	0.30	0.18	0.20	0.24	0.06

Table S17: Predictive ability of Intramuscular fat (%) for each fold at different complexity, stage and model.

Complexity	Stage	Model	Fold1	Fold2	Fold3	Fold4	Average	SD
Full	Wean	M1	0.45	0.31	0.43	0.37	0.39	0.06
Full	Off-test	M1	0.45	0.31	0.43	0.37	0.39	0.06
Full	Mid-test	M1	0.45	0.31	0.43	0.37	0.39	0.06
Full	Wean	M2	0.25	0.24	0.26	0.27	0.26	0.01
Full	Mid-test	M2	0.31	0.23	0.28	0.32	0.29	0.04
Full	Off-test	M2	0.31	0.24	0.32	0.29	0.29	0.06
Full	Wean	M3	0.45	0.33	0.43	0.38	0.40	0.05
Full	Mid-test	M3	0.45	0.31	0.42	0.39	0.39	0.06
Full	Off-test	M3	0.48	0.29	0.45	0.38	0.40	0.08
Full	Wean	M4	0.44	0.32	0.43	0.38	0.39	0.06
Full	Mid-test	M4	0.45	0.31	0.42	0.39	0.39	0.06
Full	Off-test	M4	0.47	0.29	0.45	0.38	0.40	0.08
Informatively reduced	Wean	M1	0.47	0.38	0.43	0.65	0.48	0.12
Informatively reduced	Mid-test	M1	0.47	0.38	0.43	0.65	0.48	0.12
Informatively reduced	Off-test	M1	0.47	0.38	0.43	0.65	0.48	0.12
Informatively reduced	Wean	M2	0.23	0.37	0.17	0.39	0.29	0.11
Informatively reduced	Mid-test	M2	0.29	0.33	0.19	0.42	0.31	0.1
Informatively reduced	Off-test	M2	0.23	0.31	0.31	0.43	0.32	0.08
Informatively reduced	Wean	M3	0.46	0.42	0.43	0.26	0.39	0.09
Informatively reduced	Mid-test	M3	0.48	0.41	0.41	0.29	0.4	0.08
Informatively reduced	Off-test	M3	0.47	0.4	0.43	0.27	0.39	0.09
Informatively reduced	Wean	M4	0.46	0.41	0.43	0.27	0.39	0.08
Informatively reduced	Mid-test	M4	0.47	0.4	0.41	0.29	0.39	0.08
Informatively reduced	Off-test	M4	0.47	0.39	0.43	0.27	0.39	0.09
Randomly reduced	Wean	M1	0.47	0.33	0.4	0.38	0.4	0.05
Randomly reduced	Mid-test	M1	0.47	0.33	0.4	0.38	0.4	0.05
Randomly reduced	Off-test	M1	0.47	0.33	0.4	0.38	0.4	0.05
Randomly reduced	Wean	M2	0.26	0.23	0.27	0.28	0.26	0.02
Randomly reduced	Mid-test	M2	0.28	0.22	0.28	0.29	0.27	0.04
Randomly reduced	Off-test	M2	0.28	0.24	0.32	0.28	0.28	0.04
Randomly reduced	Wean	M3	0.47	0.34	0.41	0.39	0.4	0.06
Randomly reduced	Mid-test	M3	0.47	0.34	0.4	0.39	0.4	0.06
Randomly reduced	Off-test	M3	0.47	0.34	0.43	0.38	0.41	0.06
Randomly reduced	Wean	M4	0.47	0.34	0.41	0.38	0.4	0.05
Randomly reduced	Mid-test	M4	0.46	0.34	0.4	0.39	0.4	0.05
Randomly reduced	Off-test	M4	0.47	0.34	0.42	0.38	0.4	0.06

Table S18: Predictive ability of Minolta a* for each fold at different complexity, stage and model.

Complexity	Stage	Model	Fold1	Fold2	Fold3	Fold4	Average	SD
Full	Wean	M1	0.27	0.24	0.27	0.32	0.28	0.03
Full	Mid-test	M1	0.27	0.24	0.27	0.32	0.28	0.03
Full	Off-test	M1	0.27	0.24	0.27	0.32	0.28	0.03
Full	Wean	M2	0.22	0.22	0.22	0.26	0.23	0.02
Full	Mid-test	M2	0.26	0.25	0.24	0.28	0.26	0.02
Full	Off-test	M2	0.27	0.26	0.29	0.31	0.28	0.02
Full	Wean	M3	0.25	0.23	0.26	0.31	0.26	0.03
Full	Mid-test	M3	0.28	0.25	0.27	0.32	0.28	0.03
Full	Off-test	M3	0.28	0.27	0.31	0.33	0.30	0.03
Full	Wean	M4	0.25	0.24	0.26	0.31	0.27	0.03
Full	Mid-test	M4	0.28	0.25	0.27	0.32	0.28	0.03
Full	Off-test	M4	0.29	0.27	0.31	0.33	0.30	0.03
Informatively reduced	Wean	M1	0.21	0.23	0.22	0.62	0.32	0.20
Informatively reduced	Mid-test	M1	0.21	0.23	0.22	0.62	0.32	0.20
Informatively reduced	Off-test	M1	0.21	0.23	0.22	0.62	0.32	0.20
Informatively reduced	Wean	M2	0.13	0.41	0.12	0.29	0.24	0.14
Informatively reduced	Mid-test	M2	0.21	0.4	0.13	0.44	0.3	0.15
Informatively reduced	Off-test	M2	0.20	0.44	0.32	0.47	0.36	0.12
Informatively reduced	Wean	M3	0.17	0.18	0.20	0.26	0.20	0.04
Informatively reduced	Mid-test	M3	0.25	0.24	0.2	0.31	0.25	0.05
Informatively reduced	Off-test	M3	0.22	0.25	0.26	0.28	0.25	0.03
Informatively reduced	Wean	M4	0.18	0.16	0.21	0.28	0.21	0.05
Informatively reduced	Mid-test	M4	0.25	0.23	0.21	0.30	0.25	0.04
Informatively reduced	Off-test	M4	0.22	0.22	0.26	0.29	0.25	0.03
Randomly reduced	Wean	M1	0.26	0.25	0.26	0.32	0.27	0.01
Randomly reduced	Mid-test	M1	0.26	0.25	0.26	0.32	0.27	0.01
Randomly reduced	Off-test	M1	0.26	0.25	0.26	0.32	0.27	0.01
Randomly reduced	Wean	M2	0.24	0.24	0.24	0.28	0.25	0.00
Randomly reduced	Mid-test	M2	0.25	0.25	0.23	0.28	0.25	0.01
Randomly reduced	Off-test	M2	0.24	0.25	0.25	0.27	0.25	0.01
Randomly reduced	Wean	M3	0.26	0.24	0.26	0.32	0.27	0.01
Randomly reduced	Mid-test	M3	0.26	0.25	0.25	0.32	0.27	0.01
Randomly reduced	Off-test	M3	0.26	0.26	0.26	0.31	0.27	0.01
Randomly reduced	Wean	M4	0.26	0.24	0.26	0.32	0.27	0.02
Randomly reduced	Mid-test	M4	0.26	0.25	0.26	0.32	0.27	0.01
Randomly reduced	Off-test	M4	0.26	0.26	0.26	0.31	0.27	0.01

Table S19: Predictive ability of Minolta b* for each fold at different complexity, stage and model.

Complexity	Stage	Model	Fold1	Fold2	Fold3	Fold4	Average	SD
Full	Wean	M1	0.28	0.42	0.27	0.32	0.32	0.07
Full	Mid-test	M1	0.28	0.42	0.27	0.32	0.32	0.07
Full	Off-test	M1	0.28	0.42	0.27	0.32	0.32	0.07
Full	Wean	M2	0.26	0.39	0.26	0.30	0.30	0.06
Full	Mid-test	M2	0.26	0.39	0.28	0.28	0.30	0.06
Full	Off-test	M2	0.26	0.43	0.29	0.33	0.33	0.07
Full	Wean	M3	0.27	0.41	0.26	0.32	0.32	0.07
Full	Mid-test	M3	0.27	0.41	0.27	0.3	0.31	0.07
Full	Off-test	M3	0.28	0.45	0.29	0.35	0.34	0.08
Full	Wean	M4	0.28	0.41	0.26	0.32	0.32	0.07
Full	Mid-test	M4	0.28	0.41	0.28	0.30	0.32	0.06
Full	Off-test	M4	0.28	0.46	0.28	0.35	0.34	0.09
Informatively reduced	Wean	M1	0.32	0.36	0.35	0.51	0.39	0.09
Informatively reduced	Mid-test	M1	0.32	0.36	0.35	0.51	0.39	0.09
Informatively reduced	Off-test	M1	0.32	0.36	0.35	0.51	0.39	0.09
Informatively reduced	Wean	M2	0.1	0.47	0.12	0.34	0.26	0.18
Informatively reduced	Mid-test	M2	0.19	0.5	0.22	0.4	0.33	0.15
Informatively reduced	Off-test	M2	0.12	0.57	0.19	0.46	0.34	0.21
Informatively reduced	Wean	M3	0.19	0.34	0.14	0.26	0.23	0.09
Informatively reduced	Mid-test	M3	0.23	0.36	0.2	0.23	0.26	0.07
Informatively reduced	Off-test	M3	0.18	0.42	0.15	0.28	0.26	0.12
Informatively reduced	Wean	M4	0.17	0.31	0.12	0.24	0.21	0.08
Informatively reduced	Mid-test	M4	0.21	0.34	0.18	0.21	0.24	0.07
Informatively reduced	Off-test	M4	0.19	0.41	0.16	0.28	0.26	0.11
Randomly reduced	Wean	M1	0.32	0.42	0.24	0.32	0.33	0.08
Randomly reduced	Mid-test	M1	0.32	0.42	0.24	0.32	0.33	0.08
Randomly reduced	Off-test	M1	0.32	0.42	0.24	0.32	0.33	0.08
Randomly reduced	Wean	M2	0.31	0.39	0.27	0.31	0.32	0.06
Randomly reduced	Mid-test	M2	0.3	0.39	0.27	0.3	0.32	0.06
Randomly reduced	Off-test	M2	0.32	0.4	0.28	0.32	0.33	0.07
Randomly reduced	Wean	M3	0.32	0.4	0.28	0.32	0.31	0.07
Randomly reduced	Mid-test	M3	0.31	0.42	0.24	0.31	0.31	0.07
Randomly reduced	Off-test	M3	0.33	0.43	0.26	0.33	0.32	0.08
Randomly reduced	Wean	M4	0.32	0.41	0.26	0.32	0.32	0.07
Randomly reduced	Mid-test	M4	0.31	0.42	0.25	0.31	0.31	0.07
Randomly reduced	Off-test	M4	0.33	0.42	0.26	0.33	0.33	0.08

Table S20: Predictive ability of Minolta L* for each fold at different complexity, stage and model.

Complexity	Stage	Model	Fold1	Fold2	Fold3	Fold4	Average	SD
Full	Wean	M1	0.21	0.16	0.08	0.23	0.17	0.07
Full	Mid-test	M1	0.21	0.16	0.08	0.23	0.17	0.07
Full	Off-test	M1	0.21	0.16	0.08	0.23	0.17	0.07
Full	Wean	M2	0.12	0.16	0.00	0.07	0.09	0.07
Full	Mid-test	M2	0.21	0.14	0.01	0.08	0.11	0.09
Full	Off-test	M2	0.17	0.17	0.05	0.1	0.12	0.06
Full	Wean	M3	0.17	0.16	0.07	0.22	0.16	0.06
Full	Mid-test	M3	0.22	0.15	0.08	0.20	0.16	0.06
Full	Off-test	M3	0.20	0.17	0.1	0.21	0.17	0.05
Full	Wean	M4	0.16	0.16	0.07	0.22	0.15	0.06
Full	Mid-test	M4	0.22	0.16	0.07	0.21	0.17	0.07
Full	Off-test	M4	0.20	0.17	0.10	0.21	0.17	0.05
Informatively reduced	Wean	M1	0.21	0.13	0.11	0.54	0.25	0.20
Informatively reduced	Mid-test	M1	0.21	0.13	0.11	0.54	0.25	0.20
Informatively reduced	Off-test	M1	0.21	0.13	0.11	0.54	0.25	0.20
Informatively reduced	Wean	M2	-0.01	0.33	-0.01	0.22	0.13	0.17
Informatively reduced	Mid-test	M2	0.20	0.33	0.01	0.27	0.20	0.14
Informatively reduced	Off-test	M2	0.03	0.38	0.04	0.28	0.18	0.18
Informatively reduced	Wean	M3	0.16	0.15	0.10	0.05	0.12	0.05
Informatively reduced	Mid-test	M3	0.24	0.16	0.10	0.08	0.14	0.07
Informatively reduced	Off-test	M3	0.16	0.2	0.12	0.07	0.14	0.06
Informatively reduced	Wean	M4	0.16	0.14	0.10	0.06	0.12	0.04
Informatively reduced	Mid-test	M4	0.23	0.15	0.10	0.09	0.14	0.06
Informatively reduced	Off-test	M4	0.16	0.19	0.12	0.07	0.14	0.05
Randomly reduced	Wean	M1	0.16	0.14	0.07	0.2	0.14	0.04
Randomly reduced	Mid-test	M1	0.16	0.14	0.07	0.2	0.14	0.04
Randomly reduced	Off-test	M1	0.16	0.14	0.07	0.2	0.14	0.04
Randomly reduced	Wean	M2	0.15	0.15	-0.01	0.09	0.1	0.08
Randomly reduced	Mid-test	M2	0.17	0.15	0	0.09	0.1	0.10
Randomly reduced	Off-test	M2	0.18	0.19	0.01	0.08	0.12	0.10
Randomly reduced	Wean	M3	0.15	0.12	0.06	0.19	0.13	0.04
Randomly reduced	Mid-test	M3	0.15	0.13	0.06	0.19	0.13	0.05
Randomly reduced	Off-test	M3	0.16	0.15	0.07	0.19	0.14	0.05
Randomly reduced	Wean	M4	0.15	0.13	0.06	0.19	0.13	0.04
Randomly reduced	Mid-test	M4	0.16	0.12	0.06	0.19	0.13	0.05
Randomly reduced	Off-test	M4	0.16	0.13	0.07	0.19	0.14	0.06

Table S21: Predictive ability of pH for each fold at different complexity, stage and model.

Complexity	Stage	Model	Fold1	Fold2	Fold3	Fold4	Average	SD
Full	Wean	M1	0.22	0.1	0.14	0.14	0.15	0.05
Full	Mid-test	M1	0.22	0.1	0.14	0.14	0.15	0.05
Full	Off-test	M1	0.22	0.1	0.14	0.14	0.15	0.05
Full	Wean	M2	0.13	0.13	0.15	0.1	0.13	0.02
Full	Mid-test	M2	0.14	0.12	0.17	0.11	0.14	0.03
Full	Off-test	M2	0.19	0.12	0.15	0.09	0.14	0.04
Full	Wean	M3	0.17	0.1	0.15	0.12	0.14	0.03
Full	Mid-test	M3	0.18	0.11	0.16	0.14	0.15	0.03
Full	Off-test	M3	0.23	0.11	0.15	0.12	0.15	0.05
Full	Wean	M4	0.19	0.1	0.15	0.12	0.14	0.04
Full	Mid-test	M4	0.17	0.11	0.16	0.13	0.14	0.03
Full	Off-test	M4	0.22	0.11	0.16	0.12	0.15	0.05
Informatively reduced	Wean	M1	0.17	0.05	0.04	0.52	0.20	0.22
Informatively reduced	Mid-test	M1	0.17	0.05	0.04	0.52	0.20	0.22
Informatively reduced	Off-test	M1	0.17	0.05	0.04	0.52	0.20	0.22
Informatively reduced	Wean	M2	0.09	0.30	0.05	0.25	0.17	0.12
Informatively reduced	Mid-test	M2	0.11	0.29	0.13	0.27	0.20	0.09
Informatively reduced	Off-test	M2	0.15	0.29	0.06	0.26	0.19	0.11
Informatively reduced	Wean	M3	0.16	0.10	0.03	0.08	0.09	0.05
Informatively reduced	Mid-test	M3	0.17	0.10	0.06	0.18	0.13	0.06
Informatively reduced	Off-test	M3	0.20	0.14	0.05	0.12	0.13	0.06
Informatively reduced	Wean	M4	0.16	0.07	0.03	0.08	0.08	0.05
Informatively reduced	Mid-test	M4	0.17	0.08	0.06	0.18	0.12	0.06
Informatively reduced	Off-test	M4	0.21	0.10	0.06	0.12	0.12	0.06
Randomly reduced	Wean	M1	0.19	0.11	0.16	0.13	0.15	0.05
Randomly reduced	Mid-test	M1	0.19	0.11	0.16	0.13	0.15	0.05
Randomly reduced	Off-test	M1	0.19	0.11	0.16	0.13	0.15	0.05
Randomly reduced	Wean	M2	0.16	0.11	0.14	0.11	0.13	0.03
Randomly reduced	Mid-test	M2	0.15	0.11	0.16	0.13	0.14	0.02
Randomly reduced	Off-test	M2	0.18	0.12	0.14	0.12	0.14	0.03
Randomly reduced	Wean	M3	0.17	0.11	0.15	0.13	0.14	0.04
Randomly reduced	Mid-test	M3	0.17	0.09	0.16	0.14	0.14	0.04
Randomly reduced	Off-test	M3	0.2	0.11	0.15	0.13	0.15	0.04
Randomly reduced	Wean	M4	0.16	0.11	0.15	0.13	0.14	0.04
Randomly reduced	Mid-test	M4	0.17	0.11	0.17	0.14	0.15	0.04
Randomly reduced	Off-test	M4	0.19	0.1	0.14	0.14	0.14	0.04

Table S22: Predictive ability of Subjective color score for each fold at different complexity, stage and model.

Complexity	Stage	Model	Fold1	Fold2	Fold3	Fold4	Average	SD
Full	Wean	M1	0.11	0.19	0.18	0.24	0.18	0.05
Full	Mid-test	M1	0.11	0.19	0.18	0.24	0.18	0.05
Full	Off-test	M1	0.11	0.19	0.18	0.24	0.18	0.05
Full	Wean	M2	0.06	0.11	0.00	0.00	0.04	0.05
Full	Mid-test	M2	0.02	0.06	0.05	0.04	0.02	0.03
Full	Off-test	M2	0.04	0.14	0.04	0.06	0.06	0.06
Full	Wean	M3	0.13	0.19	0.18	0.23	0.18	0.04
Full	Mid-test	M3	0.11	0.19	0.17	0.24	0.18	0.05
Full	Off-test	M3	0.13	0.21	0.16	0.24	0.18	0.05
Full	Wean	M4	0.12	0.2	0.19	0.23	0.19	0.05
Full	Mid-test	M4	0.11	0.18	0.16	0.24	0.17	0.05
Full	Off-test	M4	0.12	0.21	0.16	0.24	0.18	0.05
Informatively reduced	Wean	M1	0.17	0.15	0.18	0.58	0.27	0.21
Informatively reduced	Mid-test	M1	0.17	0.15	0.18	0.58	0.27	0.21
Informatively reduced	Off-test	M1	0.17	0.15	0.18	0.58	0.27	0.21
Informatively reduced	Wean	M2	0.01	0.42	0.05	0.2	0.16	0.19
Informatively reduced	Mid-test	M2	0.01	0.33	0.06	0.24	0.16	0.16
Informatively reduced	Off-test	M2	0.07	0.36	0.02	0.34	0.2	0.18
Informatively reduced	Wean	M3	0.17	0.19	0.19	0.17	0.18	0.01
Informatively reduced	Mid-test	M3	0.17	0.20	0.18	0.21	0.19	0.02
Informatively reduced	Off-test	M3	0.17	0.24	0.19	0.17	0.19	0.03
Informatively reduced	Wean	M4	0.17	0.17	0.19	0.17	0.18	0.01
Informatively reduced	Mid-test	M4	0.17	0.19	0.18	0.21	0.19	0.02
Informatively reduced	Off-test	M4	0.17	0.23	0.19	0.17	0.19	0.03
Randomly reduced	Wean	M1	0.14	0.17	0.21	0.25	0.19	0.02
Randomly reduced	Mid-test	M1	0.14	0.17	0.21	0.25	0.19	0.02
Randomly reduced	Off-test	M1	0.14	0.17	0.21	0.25	0.19	0.02
Randomly reduced	Wean	M2	0.02	0.09	0	0	0.03	0.05
Randomly reduced	Mid-test	M2	0.01	0.12	-0.01	0.03	0.04	0.03
Randomly reduced	Off-test	M2	0.02	0.13	0	0.01	0.04	0.07
Randomly reduced	Wean	M3	0.15	0.16	0.22	0.23	0.19	0.03
Randomly reduced	Mid-test	M3	0.14	0.17	0.21	0.25	0.19	0.03
Randomly reduced	Off-test	M3	0.14	0.18	0.22	0.24	0.2	0.02
Randomly reduced	Wean	M4	0.14	0.17	0.21	0.24	0.19	0.03
Randomly reduced	Mid-test	M4	0.13	0.17	0.2	0.25	0.19	0.02
Randomly reduced	Off-test	M4	0.14	0.18	0.22	0.24	0.2	0.03

Table S23: Predictive ability of Subjective marbling score for each fold at different complexity, stage and model.

Complexity	Stage	Model	Fold1	Fold2	Fold3	Fold4	Average	SD
Full	Wean	M1	0.23	0.36	0.17	0.35	0.28	0.09
Full	Mid-test	M1	0.23	0.36	0.17	0.35	0.28	0.09
Full	Off-test	M1	0.23	0.36	0.17	0.35	0.28	0.09
Full	Wean	M2	0.15	0.26	0.14	0.22	0.17	0.09
Full	Mid-test	M2	0.14	0.25	0.13	0.20	0.18	0.07
Full	Off-test	M2	0.18	0.25	0.15	0.16	0.19	0.08
Full	Wean	M3	0.23	0.35	0.19	0.35	0.28	0.08
Full	Mid-test	M3	0.26	0.32	0.17	0.32	0.27	0.07
Full	Off-test	M3	0.21	0.34	0.17	0.3	0.26	0.08
Full	Wean	M4	0.23	0.36	0.19	0.35	0.28	0.09
Full	Mid-test	M4	0.25	0.33	0.17	0.33	0.27	0.08
Full	Off-test	M4	0.21	0.34	0.17	0.3	0.26	0.08
Informatively reduced	Wean	M1	0.23	0.26	0.19	0.52	0.30	0.30
Informatively reduced	Mid-test	M1	0.23	0.26	0.19	0.52	0.30	0.30
Informatively reduced	Off-test	M1	0.23	0.26	0.19	0.52	0.30	0.30
Informatively reduced	Wean	M2	0.09	0.32	0.08	0.32	0.2	0.14
Informatively reduced	Mid-test	M2	0.16	0.44	0.12	0.25	0.24	0.14
Informatively reduced	Off-test	M2	0.13	0.43	0.07	0.24	0.22	0.16
Informatively reduced	Wean	M3	0.25	0.32	0.16	0.23	0.24	0.07
Informatively reduced	Mid-test	M3	0.24	0.35	0.18	0.23	0.25	0.07
Informatively reduced	Off-test	M3	0.24	0.33	0.18	0.23	0.24	0.06
Informatively reduced	Wean	M4	0.25	0.32	0.17	0.24	0.24	0.06
Informatively reduced	Mid-test	M4	0.23	0.34	0.18	0.23	0.25	0.07
Informatively reduced	Off-test	M4	0.24	0.33	0.19	0.24	0.25	0.06
Randomly reduced	Wean	M1	0.23	0.36	0.17	0.31	0.27	0.08
Randomly reduced	Mid-test	M1	0.23	0.36	0.17	0.31	0.27	0.08
Randomly reduced	Off-test	M1	0.23	0.36	0.17	0.31	0.27	0.08
Randomly reduced	Wean	M2	0.07	0.28	0.08	0.21	0.16	0.08
Randomly reduced	Mid-test	M2	0.08	0.27	0.07	0.2	0.16	0.07
Randomly reduced	Off-test	M2	0.08	0.29	0.09	0.2	0.17	0.10
Randomly reduced	Wean	M3	0.22	0.36	0.18	0.31	0.27	0.07
Randomly reduced	Mid-test	M3	0.23	0.35	0.17	0.3	0.26	0.07
Randomly reduced	Off-test	M3	0.23	0.36	0.17	0.31	0.27	0.08
Randomly reduced	Wean	M4	0.23	0.36	0.17	0.31	0.27	0.08
Randomly reduced	Mid-test	M4	0.22	0.36	0.17	0.3	0.26	0.08
Randomly reduced	Off-test	M4	0.23	0.36	0.17	0.3	0.27	0.09

Table S24: Predictive ability of Subjective firmness score for each fold at different complexity, stage and model.

Complexity	Stage	Model	Fold1	Fold2	Fold3	Fold4	Average	SD
Full	Wean	M1	0.16	0.25	0.10	0.13	0.16	0.06
Full	Mid-test	M1	0.16	0.25	0.10	0.13	0.16	0.06
Full	Off-test	M1	0.16	0.25	0.10	0.13	0.16	0.06
Full	Wean	M2	0.08	0.18	0.02	0.08	0.09	0.07
Full	Mid-test	M2	0.11	0.17	0.12	0.17	0.14	0.03
Full	Off-test	M2	0.11	0.23	0.15	0.15	0.16	0.05
Full	Wean	M3	0.13	0.21	0.08	0.11	0.13	0.06
Full	Mid-test	M3	0.15	0.20	0.15	0.18	0.17	0.02
Full	Off-test	M3	0.14	0.24	0.17	0.16	0.18	0.04
Full	Wean	M4	0.14	0.22	0.08	0.11	0.14	0.06
Full	Mid-test	M4	0.15	0.20	0.15	0.17	0.17	0.02
Full	Off-test	M4	0.15	0.26	0.17	0.16	0.19	0.05
Informatively reduced	Wean	M1	0.11	0.13	0.10	0.58	0.23	0.23
Informatively reduced	Mid-test	M1	0.11	0.13	0.10	0.58	0.23	0.23
Informatively reduced	Off-test	M1	0.11	0.13	0.10	0.58	0.23	0.23
Informatively reduced	Wean	M2	-0.05	0.33	-0.02	0.26	0.13	0.19
Informatively reduced	Mid-test	M2	0.05	0.35	0.06	0.26	0.18	0.15
Informatively reduced	Off-test	M2	0.10	0.41	0.18	0.37	0.26	0.15
Informatively reduced	Wean	M3	0.06	0.18	0.08	0.03	0.09	0.06
Informatively reduced	Mid-test	M3	0.1	0.19	0.12	0.12	0.13	0.04
Informatively reduced	Off-test	M3	0.13	0.21	0.13	0.08	0.14	0.05
Informatively reduced	Wean	M4	0.06	0.16	0.09	0.03	0.08	0.06
Informatively reduced	Mid-test	M4	0.11	0.18	0.12	0.11	0.13	0.03
Informatively reduced	Off-test	M4	0.13	0.20	0.13	0.08	0.14	0.05
Randomly reduced	Wean	M1	0.18	0.25	0.11	0.16	0.18	0.07
Randomly reduced	Mid-test	M1	0.18	0.25	0.11	0.16	0.18	0.07
Randomly reduced	Off-test	M1	0.18	0.25	0.11	0.16	0.18	0.07
Randomly reduced	Wean	M2	0.11	0.21	0.03	0.11	0.12	0.10
Randomly reduced	Mid-test	M2	0.08	0.18	0.08	0.14	0.12	0.05
Randomly reduced	Off-test	M2	0.1	0.25	0.09	0.17	0.15	0.08
Randomly reduced	Wean	M3	0.18	0.24	0.1	0.15	0.17	0.09
Randomly reduced	Mid-test	M3	0.14	0.21	0.13	0.18	0.17	0.05
Randomly reduced	Off-test	M3	0.16	0.27	0.14	0.2	0.19	0.07
Randomly reduced	Wean	M4	0.17	0.25	0.1	0.15	0.17	0.09
Randomly reduced	Mid-test	M4	0.15	0.22	0.13	0.17	0.17	0.06
Randomly reduced	Off-test	M4	0.16	0.28	0.13	0.19	0.19	0.07

Table S25: Predictive ability of Slice shear force for each fold at different complexity, stage and model.

Complexity	Stage	Model	Fold1	Fold2	Fold3	Fold4	Average	SD
Full	Wean	M1	0.22	0.15	0.16	0.20	0.18	0.03
Full	Mid-test	M1	0.22	0.15	0.16	0.20	0.18	0.03
Full	Off-test	M1	0.22	0.15	0.16	0.20	0.18	0.03
Full	Wean	M2	0.10	0.13	0.14	0.20	0.14	0.04
Full	Mid-test	M2	0.15	0.18	0.16	0.20	0.17	0.02
Full	Off-test	M2	0.16	0.14	0.16	0.17	0.16	0.01
Full	Wean	M3	0.21	0.16	0.16	0.21	0.18	0.03
Full	Mid-test	M3	0.24	0.2	0.17	0.22	0.21	0.03
Full	Off-test	M3	0.25	0.17	0.17	0.20	0.20	0.04
Full	Wean	M4	0.21	0.16	0.17	0.20	0.19	0.02
Full	Mid-test	M4	0.24	0.19	0.17	0.22	0.21	0.03
Full	Off-test	M4	0.24	0.16	0.17	0.20	0.19	0.04
Informatively reduced	Wean	M1	0.20	0.15	0.17	0.56	0.27	0.19
Informatively reduced	Mid-test	M1	0.20	0.15	0.17	0.56	0.27	0.19
Informatively reduced	Off-test	M1	0.20	0.15	0.17	0.56	0.27	0.19
Informatively reduced	Wean	M2	0.02	0.17	0.02	0.21	0.10	0.10
Informatively reduced	Mid-test	M2	0.10	0.34	-0.01	0.41	0.21	0.20
Informatively reduced	Off-test	M2	0.13	0.36	0.05	0.28	0.20	0.14
Informatively reduced	Wean	M3	0.15	0.16	0.14	0.10	0.14	0.03
Informatively reduced	Mid-test	M3	0.23	0.24	0.14	0.10	0.18	0.07
Informatively reduced	Off-test	M3	0.22	0.19	0.16	0.10	0.17	0.05
Informatively reduced	Wean	M4	0.19	0.17	0.14	0.12	0.16	0.03
Informatively reduced	Mid-test	M4	0.24	0.22	0.14	0.10	0.18	0.07
Informatively reduced	Off-test	M4	0.22	0.18	0.16	0.10	0.165	0.05
Randomly reduced	Wean	M1	0.22	0.14	0.16	0.21	0.18	0.03
Randomly reduced	Mid-test	M1	0.22	0.14	0.16	0.21	0.18	0.03
Randomly reduced	Off-test	M1	0.22	0.14	0.16	0.21	0.18	0.03
Randomly reduced	Wean	M2	0.08	0.11	0.11	0.17	0.12	0.02
Randomly reduced	Mid-test	M2	0.12	0.16	0.17	0.15	0.15	0.02
Randomly reduced	Off-test	M2	0.15	0.11	0.12	0.18	0.14	0.01
Randomly reduced	Wean	M3	0.2	0.14	0.14	0.2	0.17	0.03
Randomly reduced	Mid-test	M3	0.22	0.17	0.18	0.2	0.19	0.03
Randomly reduced	Off-test	M3	0.24	0.15	0.15	0.22	0.19	0.04
Randomly reduced	Wean	M4	0.2	0.15	0.15	0.2	0.18	0.03
Randomly reduced	Mid-test	M4	0.22	0.16	0.17	0.2	0.19	0.03
Randomly reduced	Off-test	M4	0.24	0.15	0.15	0.22	0.19	0.04

[1]M1 = Model containing genomic information only

M2 = Model containing microbiome information only

M3 = Model containing microbiome and genomic information

M4 = Model containing microbiome, genome and microbiome-by-genome information
