# Supplementary material for: Modeling host-microbiome interactions for the prediction of meat quality and carcass composition traits in swine
Source: Genet Sel Evol. 2020 Jul 29;52:41. doi: 10.1186/s12711-020-00561-7 (PMC7388461; doi:10.1186/s12711-020-00561-7)
Supplement: Supplementary file 7 — Additional file 7: Table S26. Least square means of prediction accuracy of all models averaging over all the traits. Table S27. Least square means of prediction accuracy of three stages of production averaging over all the traits. Table S28. Least square means of prediction accuracy of three complexities of model averaging over all the traits. Table S29. Least square means of prediction accuracy of interaction of stage and model averaging over all the traits. Table S30. Least square means of prediction accuracy of interaction of complexity and model averaging over all the traits. [file 12711_2020_561_MOESM7_ESM.pdf]

Table S26: Least square means of prediction accuracy of all models averaging over all the traits

Model	Least square means ± SE	Group
M0	0.15 ± 0.005	A
M1	0.21 ± 0.005	C
M2	0.23 ± 0.005	B
M3	0.24 ± 0.005	C
M4	0.25 ± 0.005	C

Table S27. Least square means of prediction accuracy of three stages of production averaging over all the traits

Stage	Least square means ± SE	Group
Wean	0.21± 0.004	A
Mid test	0.22± 0.004	B
Off test	0.23± 0.004	C

Table S28. Least square means of prediction accuracy of three complexities of model averaging over all the traits

Complexity	Least square means ± SE	Group
Full	0.20 ±0.004	A
IR	0.22 ±0.004	B
RR	0.23 ±0.004	C

Table S29 Least square means of prediction accuracy of interaction of stage and model averaging over all the traits

Interaction group	Least square means ± SE	Group
Wean:M0	0.156 ± 0.005	A
Wean:M1	0.255 ± 0.005	D
Wean:M2	0.191 ± 0.005	B
Wean:M3	0.223 ± 0.005	C
Wean:M4	0.223 ± 0.005	C
Mid test:M0	0.157 ± 0.005	A
Mid test:M1	0.220 ± 0.005	C
Mid test:M2	0.255 ± 0.005	D
Mid test:M3	0.241 ± 0.005	CD
Mid test:M4	0.239 ± 0.005	CD
Off test:M0	0.155 ± 0.005	A
Off test:M1	0.230 ± 0.005	C
Off test:M2	0.255 ± 0.005	D
Off test:M3	0.242 ± 0.005	CD
Off test:M4	0.241 ± 0.005	CD

Table S30. Least square means of prediction accuracy of interaction of complexity and model averaging over all the traits

Interaction group	Least square means ± SE	Group
IR:M0	0.156 ± 0.009	A
FULL:M0	0.156 ± 0.009	A
RR:M0	0.156 ± 0.009	A
RR:M2	0.174 ± 0.009	AB
FULL:M2	0.204 ± 0.009	BC
IR:M4	0.223 ± 0.009	CD
RR:M1	0.224 ± 0.009	CD
RR:M4	0.225 ± 0.009	CD
IR:M3	0.226 ± 0.009	CD
RR:M3	0.226 ± 0.009	CD
FULL:M1	0.235 ± 0.009	CD
FULL:M4	0.250 ± 0.009	D
FULL:M3	0.251 ± 0.009	D
IR:M2	0.263 ± 0.009	DE
IR:M1	0.305 ± 0.009	E

M1 = Model containing genomic information only
M2 = Model containing microbiome information only
M3 = Model containing microbiome and genomic information
M4 = Model containing microbiome, genome and microbiome-by-genome information
FULL = Full complexity containing all markers and OTU
IR = Informatively reduced complexity containing informatively reduced markers or OTU
RR = Randomly reduced complexity containing randomly reduced markers or OTU
Different letters in group indicates that they are different.
